# Loss of Trabid, a New Negative Regulator of the *Drosophila* Immune-Deficiency Pathway at the Level of TAK1, Reduces Life Span

**DOI:** 10.1371/journal.pgen.1004117

**Published:** 2014-02-20

**Authors:** Merennege Dilan Anush Fernando, Ilias Kounatidis, Petros Ligoxygakis

**Affiliations:** Genes and Development Laboratory, Department of Biochemistry, University of Oxford, Oxford, United Kingdom; University of California San Francisco, United States of America

## Abstract

A relatively unexplored nexus in *Drosophila* Immune deficiency (IMD) pathway is TGF-beta Activating Kinase 1 (TAK1), which triggers both immunity and apoptosis. In a cell culture screen, we identified that Lysine at position 142 was a K63-linked Ubiquitin acceptor site for TAK1, required for signalling. Moreover, Lysine at position 156 functioned as a K48-linked Ubiquitin acceptor site, also necessary for TAK1 activity. The deubiquitinase Trabid interacted with TAK1, reducing immune signalling output and K63-linked ubiquitination. The three tandem Npl4 Zinc Fingers and the catalytic Cysteine at position 518 were required for Trabid activity. Flies deficient for Trabid had a reduced life span due to chronic activation of IMD both systemically as well as in their gut where homeostasis was disrupted. The TAK1-associated Binding Protein 2 (TAB2) was linked with the TAK1-Trabid interaction through its Zinc finger domain that pacified the TAK1 signal. These results indicate an elaborate and multi-tiered mechanism for regulating TAK1 activity and modulating its immune signal.

## Author Summary

Chronic activation of immune responses results in health problems including gastrointestinal infections, metabolic imbalances and inflammatory bowel diseases that may lead to colorectal cancer. Central to this, is the balance of activation/restriction of nuclear factor-κB (NF-κB) during innate immune responses. To study signaling through NF-κB, we use the fruit fly *Drosophila melanogaster* as a genetically tractable model system that reflects human biology (due to the evolutionary conservation between innate immunity in flies and mammals), while reducing the complexity of the human disease of interest. We have found a new negative regulator of the *Drosophila* NF-κB pathway named Trabid. Its loss released the pathway and resulted in constitutive immune activation both in the gut as well as in the whole fly. This spontaneous immune activation reduced life span in the absence of infection, especially when it was combined with loss of another known negative regulator of the same pathway, a protein named Pirk. Stem cell activity in the gut in a *pirk;trabid* double mutant was found to be significantly increased, as the gut was trying to balance enterocyte loss. Trabid was acting at the level of TGF-beta Activating Kinase 1 (TAK1), which triggers both immunity and cell death.

## Introduction

Conjugation of Ubiquitin (Ub) and formation of polyubiquitin chains on proteins can stimulate the assembly of reversible, short-lived signalling centres [reviewed in 1]. The most studied of the different polyubiquitin chain types are the K48-linked and K63-linked chains. K48-linked polyubiquitin chains target a protein for proteosomal degradation while polyubiquitin chains linked by K63 function in processes including signal transduction, DNA repair and transcription, through a degradation-independent mechanism [2]. Ubiquitin-mediated signalling is particularly important for both activating and restricting the activity of nuclear factor-κB (NF-κB) during innate immune responses where deregulation leads to chronic inflammation and cancer [reviewed in 3].

In Drosophila, the IMD pathway, which shows striking similarities to the ones stimulated by members of the mammalian TNF-receptor super-family, is strongly triggered by DAP-type peptidoglycan, a cell wall component of Gram-negative bacteria and Gram-positive bacilli [reviewed in 4]. It is assumed that fragments of peptidoglycan released by these bacteria bind the peptidoglycan recognition proteins LC or LE at the cell surface or the cytosol respectively leading to their multimerization [5]. The signal is then transduced through a receptor-adaptor complex comprising Imd itself (homologous to the mammalian Receptor Interacting Protein RIP, minus the kinase domain) [6], dFADD (FAS-associated death domain protein homologue) [7], [8] and the caspase-8 homologue Dredd (death-related Ced-3/Nedd2-like protein) [9]. DREDD is K63-linked ubiquitinated by the *Drosophila* Inhibitor of apoptosis-2 (dIAP-2), which acts as an E3-ligase promoting DREDD activation [10]. DREDD then cleaves Imd thus unmasking a domain of interaction with dIAP-2 for further dIAP-2-dependent Ub-conjugation this time on Imd itself [11]. Through its RING domain, dIAP-2 ubiquitinates and stabilises Imd, which then acts like a scaffold for the recruitment of downstream components [11]. These components may include the *Drosophila* TGF-beta-activating-kinase 1 (dTAK1), together with adaptor TAK1-associated Binding Protein 2 (dTAB2) [11]–[13]. TAK1/TAB2 play a critical role in activating the *Drosophila* IκB kinase (IKK) complex and also transiently activate the c-Jun-N-terminal kinase (JNK) pathway [14]. In this IKK/JNK dichotomy, the IKK complex represents the branch of the pathway that phosphorylates the NF-κB transcription factor Relish [15]. It is probable (but not proven) that Dredd cleaves the inhibitory C-terminal domain of phosphorylated Relish helped by an IKK scaffold [15], [16] thus liberating the N-terminal portion to move to the nucleus and regulate expression of transcriptional targets such as the antimicrobial peptide (AMP) gene *diptericin* (*dipt*).

IMD signalling shows acute phase profile in terms of AMP triggering where induction is rapid following infection [17]. Negative regulation plays an important role in restricting the response both inside as well as outside of the cell in epithelia and systemic infection. Outside of the cell Peptidoglycan Recognition Proteins (PGRPs) with an amidase activity act to down-regulate the pathway following microbial sensing [18]. Inside the cell, Pirk negatively regulates the receptor PGRP-LC [19]–[21] while dUSP36 inhibits Imd itself [22] and CYLD the IKK complex [23]. Relish plays a crucial role in limiting the signal through proteosomal degradation of dTAK1 [24]. Nevertheless, it is still unclear how dTAK1 is activated although both Dredd and K63-polyubiquitin chains may be involved [25], [15]. Here we report the discovery of Trabid as a novel component of the IMD pathway and a negative-regulator of dTAK1. Trabid altered K63-linked polyubiquitination in dTAK1 through its OTU and NZF domains attenuating the immune-related but not the JNK-related signalling output of dTAK1. We found Lysine 142 of dTAK1 to be critical for its function in the pathway as the probable K63 polyubiquitin acceptor site. Further, K156 functioned as a potential K48 Ub acceptor site. In addition, dTAB2 was found to interact with Trabid and modulate dTAK1 activity through its Zinc finger domain. Together, our findings indicate an elaborate and multi-tiered process modulating dTAK1 signalling activity, during Gram-negative bacterial infection.

## Results

### Lys142 and Lys156 in dTAK1 are essential for immune signalling

**Figure 1:**
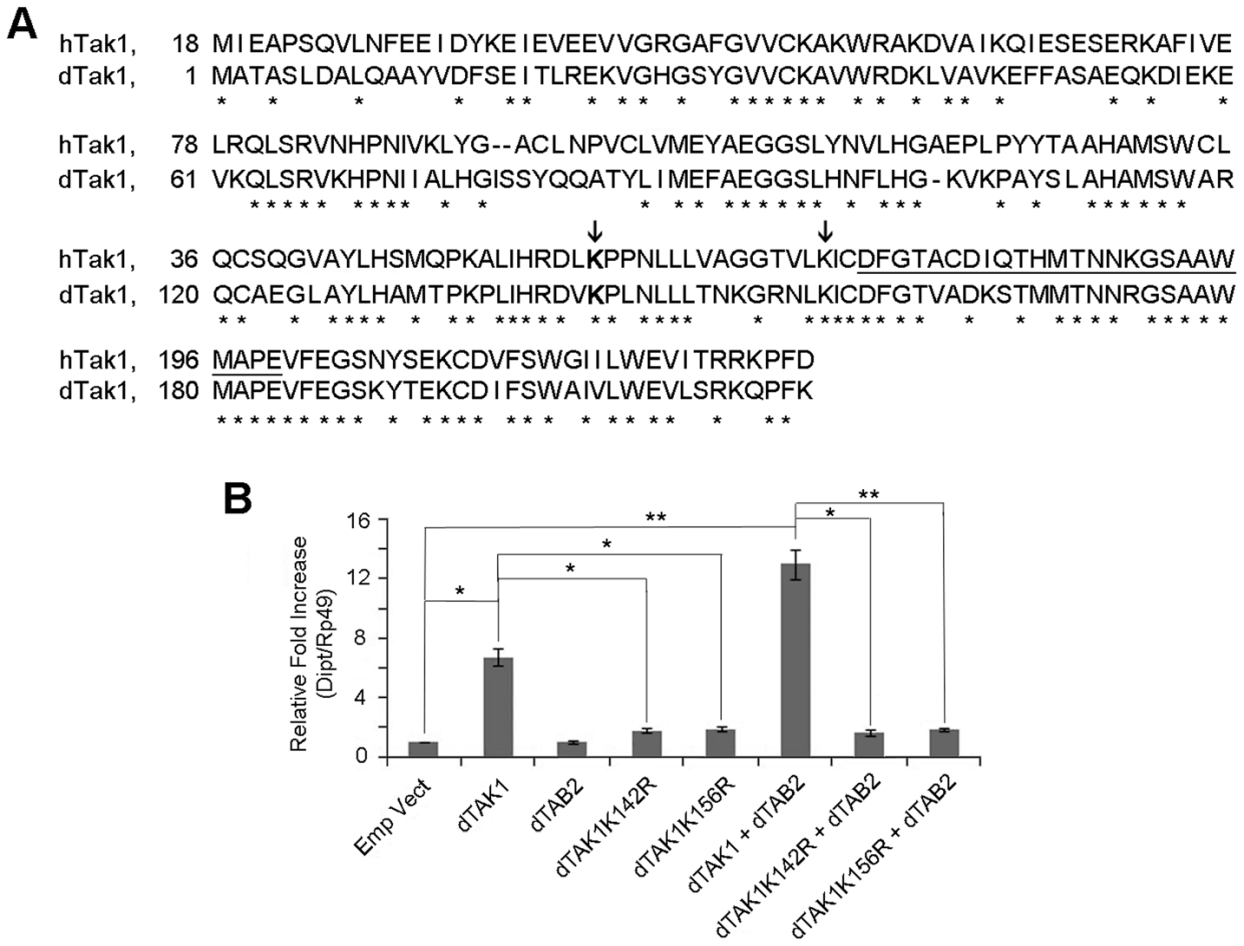
Lys 142 and Lys 156 of dTAK1 are essential for immune signalling. (A) Sequence alignment of 1 to 230 amino acids of human TAK1 (hTAK1) and *Drosophila* TAK1 (dTAK1). Lys 142 (dark grey) and 156 (light grey) are indicated (arrows) and the kinase activation loop is underlined. (B) *Drosophila* TAK1^K142R^ and TAK1^K156R^ mutants failed to activate *diptericin*. *Drosophila* TAK1 or dTAK1^K142R^ or dTAK1^K156R^ mutant was expressed along with or without dTAB2, in S2 cells in combinations shown. *Diptericin* expression was assayed 48 hrs post-transfection. Error bars represent Standard Error of 3 separate experiments. *p<0.05, **p<0.01 indicate significant value when dTAK1 wild type or dTAK1 (+dTAB2) were compared with the empty vector.

In mammals, K63-linked polyubiquitination of TAK1 at Lys158 is critical for activating several signalling cascades including Tumor Necrosis Factor alpha (TNFα), Interleukin-1beta (IL-1beta)-induced IkappaB kinase (IKK)/nuclear factor-kappaB (NF-κB) and c-Jun N-terminal kinase (JNK)/activator protein 1 (AP-1) pathway [26]. We aligned the sequences of human TAK1 (hTAK1; 1–230 amino acids) and its fruit fly orthologue (dTAK1) to determine the corresponding Lys in *Drosophila* (Figure 1A). Based on the sequence of residues around Lys158 in hTAK1, Lys142 of dTAK1 was identified as the most probable candidate. Previous studies had also implicated Lys34 and 209 as Ub acceptor sites in hTAK1 [27]. Based on sequence alignments we identified Lys194 as the *Drosophila* equivalent of Lys209 in hTAK1. However, the equivalent of Lys34 in humans is not present in *Drosophila* (Figure 1A). It was also plausible that the K63 & K48 putative Ub acceptor sites would be in close proximity to both each other and to the kinase activation domain. Hence, Lys134, 156 and 189 were also selected as likely candidates.

Mutation constructs dTAK1^K142R^-V5, dTAK1^K134R^-V5, dTAK1^K156R^-V5, dTAK1^K189R^-V5 & dTAK1^K194R^-V5 were made changing the Lys to Arg at these sites. These point mutations maintained the positive charge but could not serve as an acceptor site for Ub modification. The constructs were then screened for their ability to activate IMD immune signalling in cell culture using quantitative real time PCR (qPCR) to measure induction of *dipt* 48 hrs post-transfection. While over expression of dTAK1 activated IMD signalling as previously observed [14], concomitant overexpression with dTAB2 resulted in increased activation (Figure 1B). In this screen, dTAK1^K142R^ and dTAK1^K156R^ showed significantly reduced *dipt* induction when compared to wild-type dTAK1 while all other mutants activated *dipt* at wild type levels (Figure 1B; Figure S1).

However, the fact that the signalling capacity of dTAK1^K156R^ and dTAK1^K142R^ was significantly reduced could have been due to the two mutant proteins not folding properly and being therefore, non-functional. As a result, they would be targeted for degradation by the proteasome. If this were the case, using a proteasome inhibitor one could show increased accumulation of non-degraded mutants dTAK1^K156R^ and dTAK1^K142R^ in comparison to wild type dTAK1. A time-course expression analysis was performed after treatment with 26S proteasomal inhibitor MG132 (75 µM for 8 hrs) at concentrations that are known to block proteasome activity as previously described [16]. Results showed that expression profiles of wild type dTAK1, and those of dTAK1^K142R^ and dTAK1^K156R^ were similar, indicating that the mutant proteins were not accumulating more than wild type dTAK1 and thus were presumably folding correctly (Figure S2). Therefore, dTAK1^K142R^ and dTAK1^K156R^ were selected for further analysis with the working hypothesis that the mutated Lysines were essential for dTAK1 immune activity.

### Ubiquitination of dTAK1 is altered in the K142R mutant

**Figure 2:**
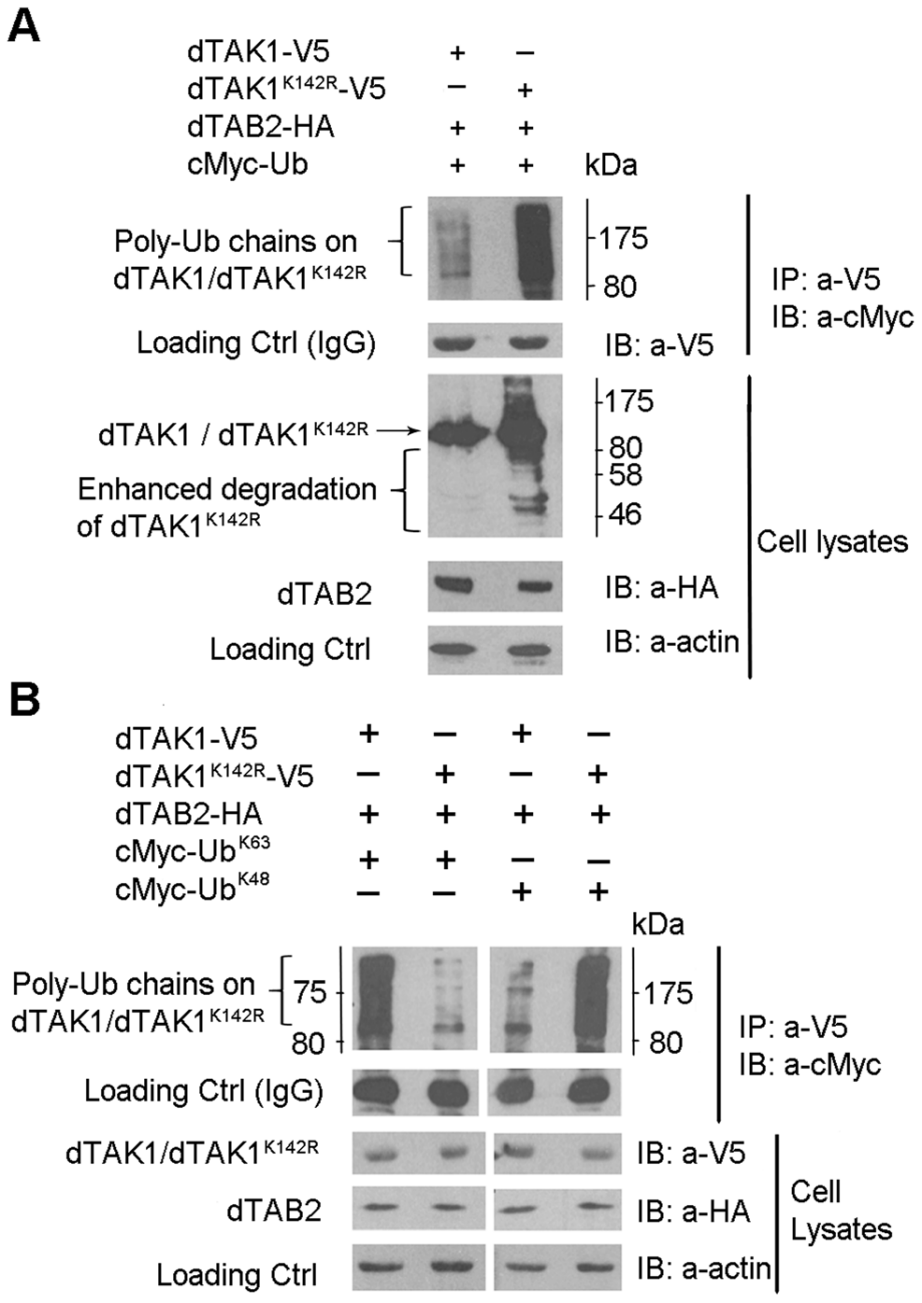
Ubiquitination profile is altered in the dTAK1^K142R^ mutant. (A) Mutant dTAK1^K142R^ showed enhanced ubiquitination. Expression vectors encoding dTAK1-V5 or dTAK1^K142R^-V5 were transfected in S2 cells along with dTAB2-HA and cMyc-Ub in combinations as shown. Cells were lysed 48 hrs post-transfection, immunoprecipitated with anti-V5, resolved on 10% SDS PAGE, and immunoblotted with anti-cMyc. Observe the enhanced degradation of dTAK1^K142R^-V5 in the cell lysate (right lane). (B) Mutant dTAK1^K142R^ showed enhanced K48-linked ubiquitination and very little K63-linked ubiquitination. Expression vectors encoding dTAK1-V5 or dTAK1^K142R^-V5 were transfected in S2 cells along with dTAB2-HA and cMyc-UbK63 (left panel) or cMyc-UbK48 (right panel) in combinations as shown. Cells were lysed 48 hrs post-transfection, immunoprecipitated with anti-V5 antibody, resolved on 10% SDS PAGE, and immunoblotted with anti-cMyc antibody. Protein size markers (NEB) are depicted adjacent to the top panels with values given in kDa.

We next sought to determine whether there was a difference in the ubiquitination profile of dTAK1 and dTAK1^K142R^ mutant. Co-overexpression of hTAK1 and TAB1 in cell culture results in hTAK1 polyubiquitination [26]. This assay was modified for *Drosophila* S2 cells. Expression vectors encoding for C-terminally V5 tagged dTAK1 or dTAK1^K142R^ were co-transfected with dTAB2-HA and cMyc-Ub into S2 cells. Cells were lysed 48 hrs post-transfection, immunoprecipitated with anti-V5 antibody, resolved on 10% SDS PAGE and immunoblotted with anti-cMyc antibody (Figure 2A). In contrast to human TAK1, where mutation of the Lys158 Ub acceptor site to Arginine resulted in failure of TGF-β-induced ubiquitination [26], [27], ubiquitination of dTAK1^K142R^ was dramatically increased. Examination of the cell lysates showed greater degradation of dTAK1^K142R^ when compared with wild type dTAK1 (Figure 2A, see dTAK1/dTAK1^K142R^ panel).

### Lys 142 is the probable K63 Ub acceptor site in dTAK1

We then analysed the linkage type of these polyubiquitination chains to distinguish whether they were K48 or K63-linked chains. We co-transfected dTAK1-V5 or dTAK1^K142R^-V5 and dTAB2-HA together with cMyc-Ub mutants having only one Lys residue at position 48 or 63 (Ub^K48^ or Ub^K63^; see experimental procedures). Wild type dTAK1 showed primarily K63-linked polyubiquitination (Figure 2B). As expected from the degradation seen in the cell lysates of Figure 2A, elimination of Lys142 severely compromised the ability of dTAK1 to form K63-linked polyubiquitination chains, although it retained the ability to form K48-linked chains (Figure 2B). Therefore, in agreement with results on human TAK1, there appeared to be two separate Ub acceptor sites for K48 and K63-linked polyubiquitination chains in *Drosophila* TAK1 with Lys142 being the probable K63 Ub acceptor site.

### Lys 156 is the probable K48 Ub acceptor site

**Figure 3:**
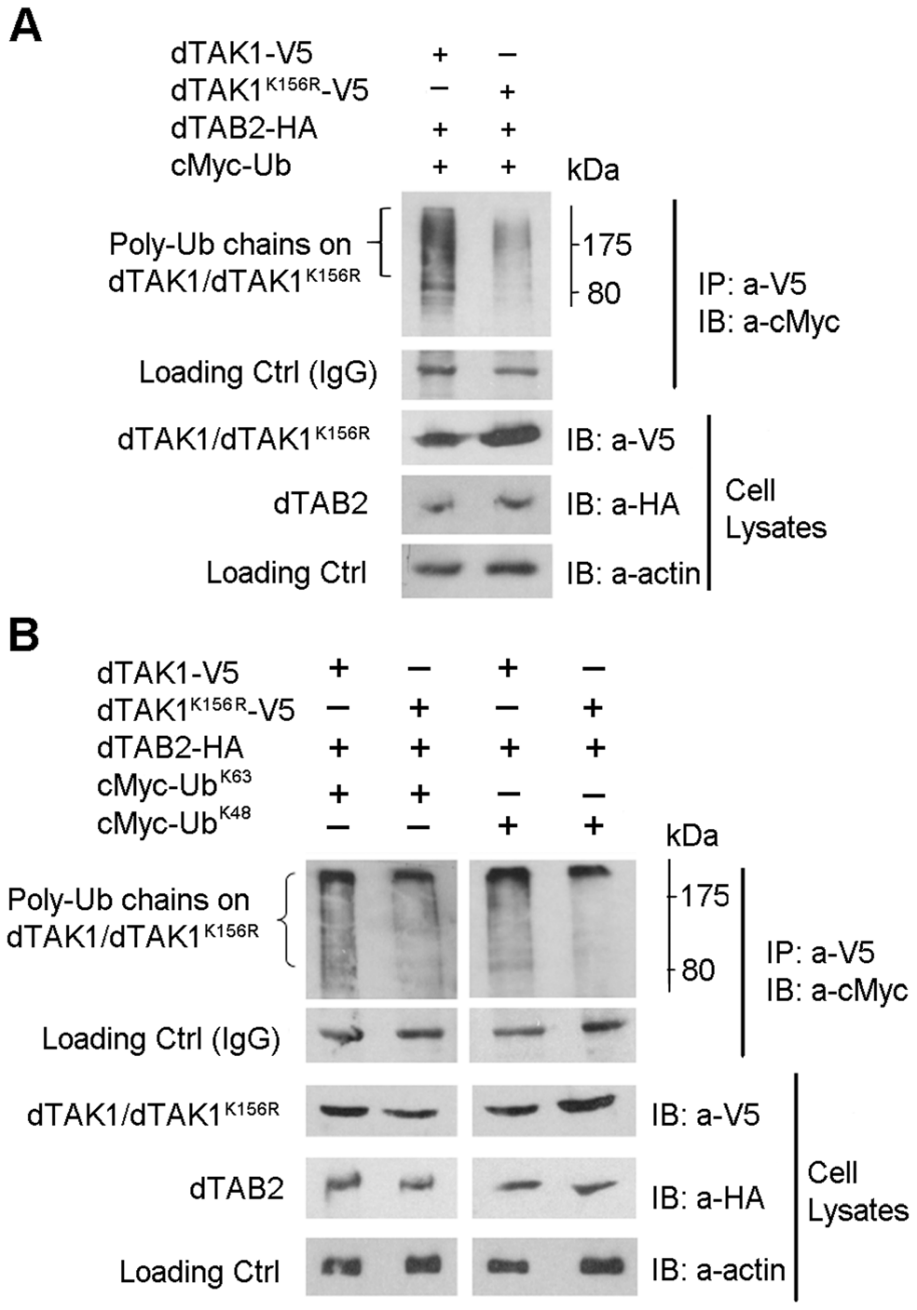
Decreased ubiquitination in the dTAK1^K156R^ mutant. (A) Mutant dTAK1^K156R^ showed decreased polyubiquitination when compared with dTAK1. Expression vectors encoding dTAK1-V5 or dTAK1^K156R^ -V5 were transfected along with dTAB2-HA and cMyc-Ub in combinations shown in S2 cells. Cells were lysed 48 hrs post-transfection, immunoprecipitated with anti-V5 antibody, resolved on 10% SDS PAGE, and immunoblotted with anti-cMyc antibody. (B) K48-linked polyubiquitination was decreased in the dTAK1^K156R^ mutant while K63-linked polyubiquitination was somewhat reduced. Expression vectors encoding dTAK1-V5 or dTAK1^K156R^-V5 were transfected along with dTAB2-HA and cMyc-Ub, cMyc-UbK63 (left panel) or cMyc-UbK48 (right panel) in combinations shown in S2 cells. Cells were lysed 48 hrs post-transfection, immunoprecipitated with anti-V5 antibody, resolved on 10% SDS PAGE, and immunoblotted with anti-cMyc antibody. Protein size marker is depicted adjacent to the top panels with values in kDa.

We then sought to determine the K48 Ub acceptor site and asked whether there was a difference in the Ub profile of dTAK1 and dTAK1^K156R^. Expression vectors encoding dTAK1-V5 or dTAK1^K156R^-V5 were transiently transfected together with dTAB2-HA and cMyc–Ub into S2 cells and Ub assays performed as above. Results showed that overall ubiquitination in the K156R mutant was greatly reduced in comparison to wild-type dTAK1 (Figure 3A).

We next identified, which type of polyubiquitination (i.e. whether K48 or K63) had been affected by the K156R mutation. Expression vectors encoding dTAK1-V5 or dTAK1^K156R^-V5 were transiently transfected together with dTAB2-HA and either cMyc – Ub^K48^ or cMyc-Ub^K63^ into S2 cells, in combinations shown and Ub assays performed (Figure 3B). Results showed that K48-linked polyubiquitination was significantly diminished in dTAK1^K156R^. Moreover, K63-linked polyubiquitination was not reduced in dTAK1^K156R^ (Figure 3B). The caveat in the above is that results have been obtained through overexpressing the relevant proteins and looking at ubiquitination. However, these data suggest that K63-linked ubiquitination must precede TAK1 activation whereas 48K-linked ubiquitination must in its turn follow to dampen the signal. We have observed such a sequence of events in a time-course experiment where we precipitated endogenous TAK1 with an antibody against it [15] and blotted for cMyc-Ub^K63^ or cMyc-Ub^K48^ following addition of peptidoglycan (PG). Our results show that 2 h following challenge with PG from *E. coli* there is a bias towards K63-linked ubiquitination, which is gradually shifted towards K48-linked ubiquitination at the end of the 6 h time course (Figure S3A). Moreover, activation of AMP-related immune responses with PG was comparable to TAK1 overexpression through transient transfection (Figure S3B).

### Trabid – a novel negative regulator of the IMD pathway

As implied from the results presented so far, K63-ubiquitination played a critical role in activating dTAK1. It follows therefore, that de-ubiquitination would be required for terminating the dTAK1 signal. In mammals, the zinc-finger protein A20 (also known as TNFAIP3) has been identified as negative regulator of NF-κB transcription factors in both TNF and IL-1 signalling through its deubiquitinating (DUB) and ubiquitin editing functions. In this model, K63-linked chains are cleaved and re-arranged to form K48-linked Ub chains, thereby tagging the protein for proteosomal degradation [28], [ reviewed in 29].

**Figure 4:**
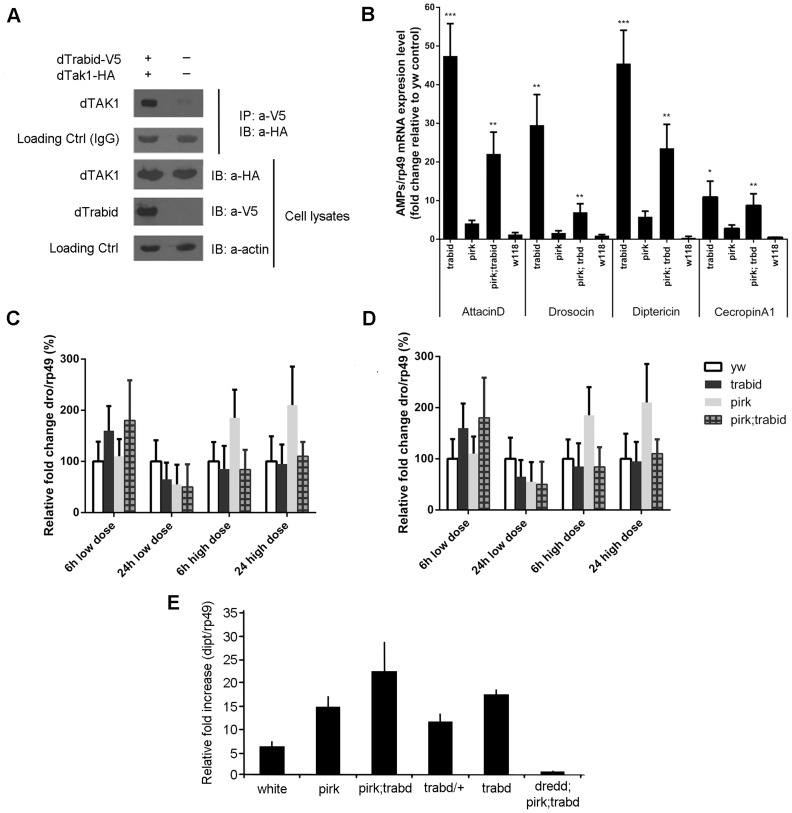
*Drosophila* Trabid binds to dTAK1 and negatively regulates IMD signalling. (A) *Drosophila* Trabid interacted with dTAK1. *Drosophila* TAK1-HA was co-transfected with or without dTrabid-V5 in S2 cells, immunoprecipitated with anti-V5 antibody, resolved on 10% SDS PAGE and immunoblotted with anti-HA antibody. (B) AMP gene expression was highly elevated in *trabid* mutants in the absence of infection. Expression levels of various AMPs (*attacinA; drosocin; diptericin; cecropinA1*) were checked in whole flies (3–6 days old) using qPCR compared with *yw* as control for the genetic background (wild type) of *pirk* and *trbd* [30], [31]. We also used the *w^118^* strain as an additional control (to check the range between two independent controls). Error bars represent the Standard Error of 3 separate experiments. *p<0.05, **p<0.01, ***p<0.001 indicate significant value when *trbd* mutants are compared with the *yw* control. Expression of *diptericin* (C) and *drosocin* (D) was not significantly different in *yw*, *pirk*, *trabid* or *pirk; trbd* mutants in response to systemic infection. *yw* (wild type), *trbd, pirk and pirk; trbd* flies, were injected with two doses of *Ec1106* (low dose ≈400 cells, high dose ≈4000 cells) and AMP levels assayed after 6 and 24 hrs using qPCR. Error bars represent the Standard Error of 3 separate experiments. Expression in *yw* was set as 100%. Calculations using paired t-test showed that the difference between mutants and *yw* was not significant (E) Drosophila Trabid negatively regulated IMD signalling in a dose-dependent manner during gut infection. *yw* (wild type), *dredd, trbd/+, trbd, pirk; trbd and dredd; pirk; trbd flies*, were fed with Ecc15 and *diptericin* levels assayed after 24 hrs using qPCR. Error bars represent the Standard Error of 3 separate experiments. The Dpt/Rp49 ratio was normalized with the level obtained in *yw* guts. All the mutants showed statistically significant increase of diptericin levels (p<0.05). Of note that, any up-regulation over and above the wild type levels was suppressed in a *dredd* background in agreement with the hypothesis that *trbd* was negatively regulating the Imd pathway (p***<0.001 when *pirk; trbd* and *dredd; prik; trbd* were compared as determined by Student's t-Test). *p<0.05 indicates significance value when *trbd/+* and *trbd* were compared as determined by Student's t-test.

Trabid (Trbd) was originally discovered as a positive regulator of both the mammalian and *Drosophila* Wnt pathway with a remarkable preference for binding to, and cleaving, K63-linked ubiquitin chains [30]. Trbd is also a representative of the A20 OTU family in fruit flies [30]. To determine whether Trbd also functioned in IMD signalling, we tested for its interaction with dTAK1. Co-immunoprecipitation assays showed that dTrbd bound to dTAK1 (Figure 4A). Trbd also bound TAB2 as shown by relevant co-immuno-precipitation experiments (see below).

We then explored the *in vivo* effects on IMD signalling of a *trbd* deletion generated by homologous recombination [30]. We assayed AMP expression in 3–6 days old whole animals without an infection. We used flies homozygous for the *trbd* deletion; flies homozygous for a mutation in *pirk* [*pirk^EY00723^*, ref 21]; *yellow-white* (*yw*) flies as the genetic background used for both the initial *trbd* targeting construct [30] and by Gene Disruption Project [31], which generated *pirk^EY00723^*; flies mutant for both *trbd* and *pirk*; *white^118^* flies (*w^118^*) as an additional control. We observed a significant increase in expression of *attacinD, drosocin* (*dro*), *dipt* and *CecropinA1* in both the *trbd* and *pirk; trbd* flies relative to the *yw* and *w^1188^* controls as well as to the *pirk* single mutant (Figure 4B). This meant that removal of *trbd* (or both *pirk* and *trbd*) would increase the levels of AMPs in a systemic fashion leading to a chronic response in the absence of infection. Injection of bacteria did not increase AMPs further and following systemic infection with *E. coli 1106* the levels of *dipt* or *dro* gene expression between control and *trbd* or *pirk;trbd* flies were statistically inseparable (Figure 4C; Figure 4D). No further increase in whole fly AMP levels following systemic infection was observed in mutants of other negative regulators (e.g. CYLD, Nubbin) [23], [32]. Further increases maybe tissue specific (gut; see below) and would therefore fail to be detected in whole fly preps.

The *Drosophila* IMD pathway is also the mediator of local immune responses in the gut. In contrast to whole animals, we observed that the gut of heterozygous *trbd* flies showed a 2-fold increased expression of *dipt* over and above the wild type levels of induction following infection with *Erwinia carotovora carotovora* (*Ecc15*) and assaying using qPCR 24 h later (Figure 4E). Loss of both copies of *trbd* resulted in *dipt* induction 3 times as much as the wild type control. Finally, concomitant loss of *pirk* and *trabid* led to a 5-fold induction of *dipt* over and above wild type activation levels (Figure 4E). This was suppressed in a *dredd* mutant background (Figure 4E). Over-activation of *dipt* was not due to a delay in bacterial clearance as exemplified by measuring Colony forming Units (CFUs) following oral *Ecc15* infection. Clearance in *trbd* and *pirk; trbd* mutants was statistically indistinguishable from wild type controls or *pirk* single mutants (Figure S4). Interestingly, bacterial clearance following systemic infection in *trbd* and *pirk; trbd* was statistically significantly faster than wild type and *pirk* flies using both a low (approx. 400 cells; Figure S5A) and a high (approx. 4000 cells; Figure S5B) dose of *E. coli 1106*. These differences were just below the limit of statistical significance following *Ecc15* systemic infection with the same doses (Figure S5C and S5D). Nevertheless, the *E. coli 1106* result correlated with the observation that *trbd* and *trbd;pirk* flies had a much higher level of AMPs to begin with (Figure 4B), suggesting a protective effect.

**Figure 5:**
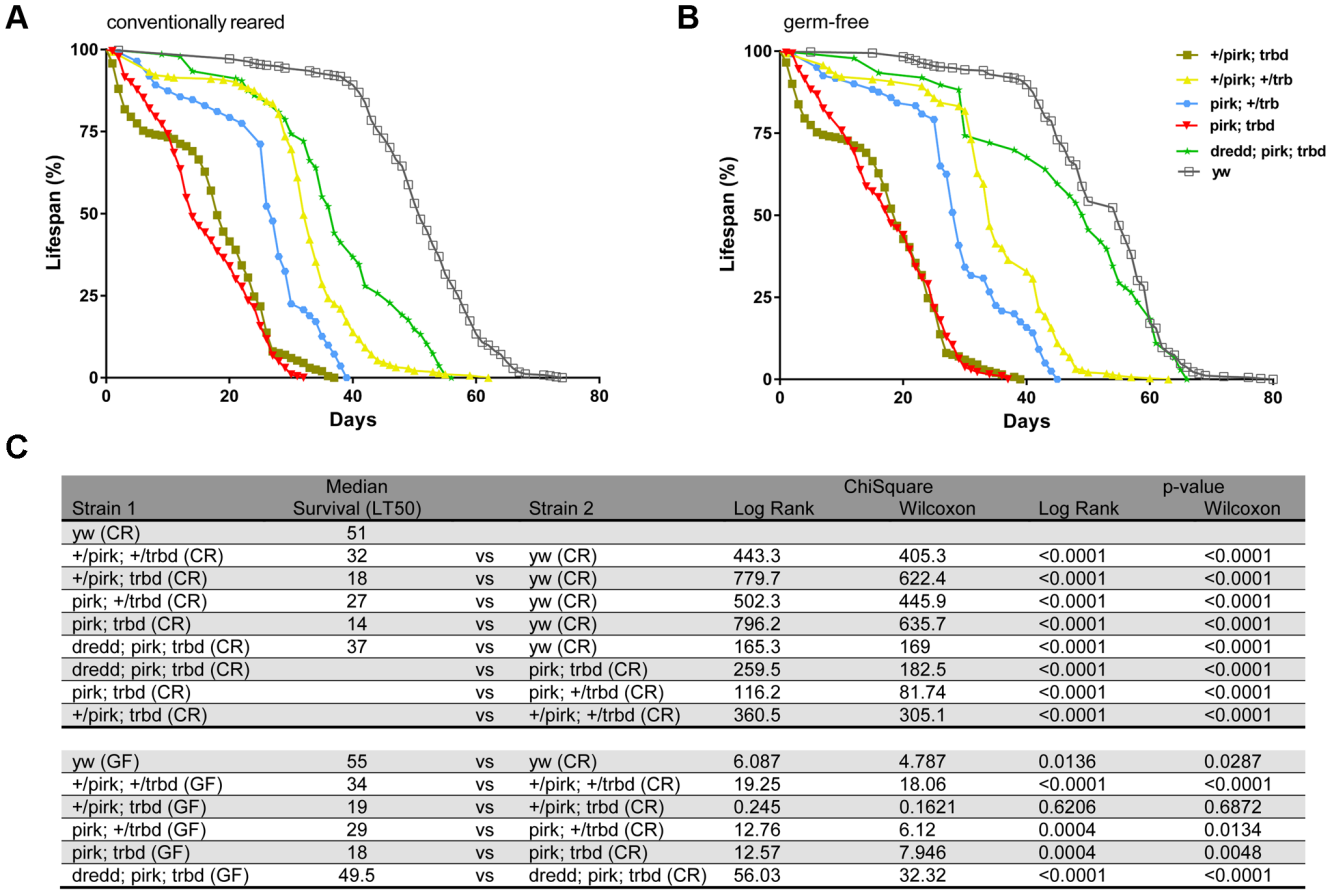
Loss of Trabid reduces life span. (A) Lifespan analysis of conventionally reared *trbd* mutans. Using the Log-Rank and Wilcoxon tests, life span analysis of flies revealed a statistically significant decrease in the survival rate of *trbd* and *trbd; pirk* mutant flies in comparison to *yw* flies. (p<0.0001 for both tests). Consistent with the assumption that decreased lifespan was a consequence of an excessive activation of Imd signalling *dredd; pirk; trbd* flies were significantly longer lived than the *pirk; trbd* double mutants (p<0.0001 for both tests). Each survival curve was a result of 12 independent experiments (360 flies in total per genotype; see experimental procedures) (B) Lifespan analysis of *trbd* mutants reared in germ-free conditions. Growing *trbd* or *pirk; trbd* flies in germ free conditions did not ameliorate life span as they were statistically inseparable to their normally grown siblings (p = 0.6206/0.6872 and p = 0.0004/0.0048 respectively). Each survival curve was a result of 12 independent experiments (360 flies in total per genotype; see experimental procedures). (C) Statistical analysis of life span results in A and B. Experimental and control populations are compared using Log-Rank and Wilcoxon tests (ChiSquare and p-values). All the analysis was performed using GraphPad Prism statistical software. See materials and methods for protocol used for germ free flies.

This potential protective effect however, had a cost. We found that deletion of *trbd* severely compromised the life span of flies (in the absence of infection). Our results are shown in Figure 5. 50% of flies heterozygous for both *pirk* and *trbd* survived the 30-day mark (LT_50_ = 32; Figure 5A and 5C). A similar effect was observed in flies deficient for *pirk* in a *trbd* heterozygous background (LT_50_ = 27; Figure 5A and 5C). Just deleting *trbd* (in a *pirk* heterozygous background) had serious consequences, as 50% of flies were dead by 18 days (LT_50_ = 18; Figure 5A and Figure 5C). More significantly however, the double mutant *pirk; trbd* had a dramatic reduction in life span compared to either single mutant (*+/pirk; trbd* or *pirk; +/trbd*) or double heterozygote (*+/pirk; +/trbd*) since 50% of flies were dead before the 14-day mark (LT_50_ = 14; Figure 5A and 5C). This phenomenon was suppressed in *dredd; pirk; trbd* flies (LT_50_ = 37) where IMD was inactive (Figure 5A; Figure 5C).

Interestingly, there was no statistically significant change (LT_50_ = 14 to LT_50_ = 18) when *pirk; trbd* flies were grown on germ-free conditions showing that it was largely the host rather than a disturbance in microbiota that was the cause for the reduction in life span (Figure 5B; Figure 5C). Crucially for this assumption, flies that had a blocked IMD pathway (*dredd; pirk; trbd*) lived as long as wild type (*yw*) flies in germ-free conditions (Figure 5B; Figure 5C).

The above result meant that chronic activation of IMD in the absence of infection (seen in Figure 4B) was the cause of life span reduction. Moreover, gut homeostasis was disrupted. Upd3 is a ligand secreted by stressed enterocytes, which activates the JAK-STAT pathway in intestinal stem cells to promote both their division and differentiation [33]–[35]. In comparison to wild type flies, we observed a significantly higher level of JAK-STAT activity in the guts of unchallenged *trbd* and even more in *pirk;trbd* flies as monitored by the expression of *upd3* and the JAK-STAT target gene *Socs36E* in qPCR assays (Figure S6A; Figure S6B, respectively). The presence of *dredd* fully suppressed this elevated JAK-STAT activity observed in *pirk;trbd* flies, demonstrating that excessive Imd pathway activation must cause gut damage, which in turn induces epithelium renewal (Figure S6A; Figure S6B). From the above experiments we concluded therefore, that 1) dTrbd physically interacted with dTAK1, 2) negatively regulated the IMD signal *in vivo* and 3) its deletion had an impact on life span and gut homeostasis due to chronic activation of immune signalling. Moreover, reduction of life span in *trbd* deletion flies was enhanced when Pirk, a known negative regulator of the IMD pathway was also mutated.

### *Drosophila* Trabid de-ubiquitinates the K63-linked chains of dTAK1

**Figure 6:**
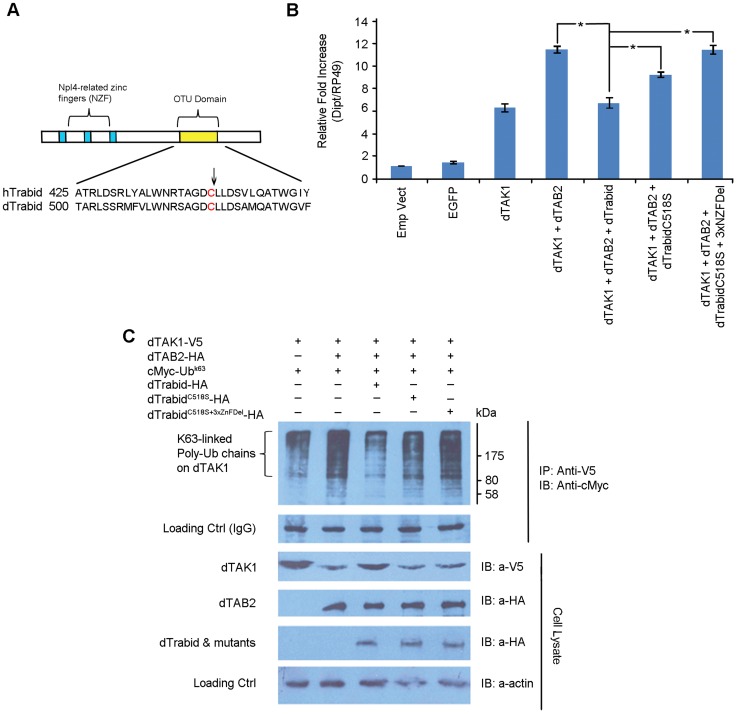
*Drosophila* Trabid significantly decreases K63-linked polyubiquitination in dTAK1 through its catalytic C518 and NZF domains. (A) Schematic representation of dTrabid and sequence alignment of human and *Drosophila* Trabid in the OTU domain. The putative catalytic residue is in red & is indicated by an arrow. (B) Point mutations in C518 and the 3 NZF domains significantly affect dTrabid function in Imd signalling. Expression vectors containing dTrabidC518S, dTrabidC518S+3xNZFDel, dTAK1 and dTAB2 were co-transfected in S2 cells in the combinations shown and *diptericin* and *puckered* expression was assayed by qPCR 48 hrs post-transfection. Results represent mean values of 3 independent experiments and the error bars SEM. Asterisk (*) indicates significance value of the results when compared to dTAK1+dTAB2 as determined by Student's t-Test (t = 9.8004, *p*<0.025). (C) *Drosophila* Trabid reduces K63-linked ubiquitination in dTAK1. Expression vectors containing C-terminally HA-tagged dTrabid, dTrabidC518S or dTrabidC518S+3xNZFDel, along with dTAK1-V5, dTAB2-HA and cMyc-UbK63 were co-transfected in S2 cells in the combinations shown. Cells were lysed 48 hrs post-transfection, immunoprecipitated with anti-V5 antibody, resolved on 10% SDS PAGE and immunoblotted with anti-cMyc peroxidise. Protein size markers (NEB) are depicted adjacent to top panels with values given in kDa.

A20 proteins (including Trbd) belong to the ovarian tumour (OTU) family of DUBs. The OTU is a conserved cysteine protease domain that possesses DUB activity [reviewed in 36]. Trbd possesses three N-terminal NZF (Npl4 zinc finger) domains and a C-terminal OTU domain, which shows DUB activity with a preference for K63-linked ubiquitin [30]. Further, two or more NZF domains are required for binding to K63-linked Ub chains. The catalytic residue in the OTU domain in humans is C443. Sequence alignment of hTrbd and dTrbd showed that C518 was most probably the corresponding catalytic residue in *Drosophila* (Figure 6A). Two constructs were made: dTrbd^C518S^ (where C518 was changed to S) and dTrbd^C518S+3xNZFDel^, where along with C518S the first Cys residue of each of the 3 NZF domains namely, C13, C94 and C238, were mutated to Ala. To determine their functional importance, these mutations, dTAK1 and dTAB2 were co-transfected along with dTrbd^C518S^ and dTrbd^C518S+3xNZFDel^ in S2 cells and relative *dipt* expression assayed using qPCR. As expected, over-expression of dTrbd significantly decreased the TAK1/TAB2-mediated *dipt* induction although it did not completely abrogate it (Figure 6B). The C518S mutation substantially relieved the suppressive effect of wild type Trbd on *dipt* expression levels, with the C518S+3xNZFDel showing no suppression whatsoever (Figure 6B). Expression levels of *puckered* (a target of the JNK cascade also induced through TAK1/TAB2 activity) revealed that the pathway was not affected, indicating that dTrbd functioned solely in the IMD pathway (Figure S7). It has been suggested that it is Plenty of SH3 (POSH), which terminates the JNK-related dTAK1 signal [37].

To determine the relative contribution of the OTU and NZF domains in the ability of dTrbd to cleave K63-linked Ub chains [30] we tested the overexpression of dTrbd as well as dTrbd^C518S^ and dTrbd^C518S+3xNZFDel^ on dTAK1 K63-linked ubiquitination. As expected, dTrbd substantially reduced K63-linked ubiquitination of dTAK1 when co-transfected with dTAB2 (Figure 6C). Ubiquitination was moderately affected by dTrbd^C518S^ and not at all by dTrbd^C518S+3xNZFDel^ (Figure 6C). We also tested the effects of dTrbd, dTrbd^C518S^ and dTrbd^C518S+3xNZFDel^ on K48-linked ubiquitination. Results showed that K48-ubiquitination on TAK1, was not affected by dTrbd wild type, or dTrbd mutants (Figure S8).

### TAB2 modulates TAK1 signalling and interacts with Trbd

We finally wanted to explore the tripartite relationship between TAK1, TAB2 and Trbd. In mammals, TAB2 and TAB3 function as adaptors, which link TRAF2 and TRAF6 to TAK1, facilitating complex formation and activation of TAK1 in IL-1 and TNF-induced NF-kB activation [38], [39]. Both TAB2 and TAB3 contain a highly conserved C-terminal novel zinc finger domain, which binds preferentially to K63-linked polyubiquitin chains. Mutations in this domain abolish the ability of TAB2 and TAB3 to bind polyubiquitin chains, as well as their ability to activate TAK1 [39], [40].

**Figure 7:**
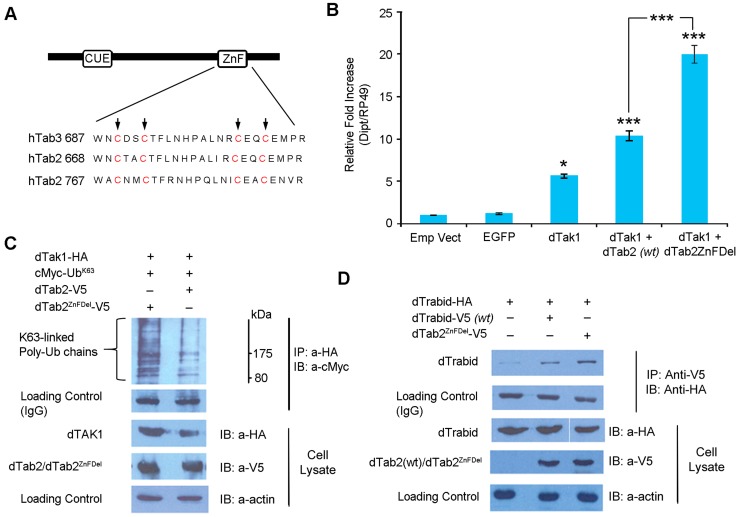
*Drosophila* TAB2 ZnF domain regulates IMD signalling. (A) Sequence alignment of the C-terminal ZnF domain of hTAB2, hTAB3 and dTAB2. Conserved Cys residues are in red and indicated by arrows. (B) IMD signalling is significantly increased in dTAB2^ZnFDel^ mutant. S2 cells were co-transfected with expression vectors in combinations shown and *diptericin* expression was assayed 48 hrs post-transfection by qPCR. Results are mean values from 3 independent experiments and the error bars represent the Standard Error. Single, and triple asterisk indicates significance value of the result when compared to EGFP as determined by Student's t-Test (t = 17.8435, p<0.0025; t = 6.2838, p<0.025 respectively). However, dTAK1+dTAB2 was significantly lower than dTAK1+dTAB2^ZnFDe**l**^ when compared. (C) Mutation of dTAB2 ZnF stabilises dTAK1 and increases its K63-linked ubiquitination. *Drosophila* TAK1-HA was co-transfected with cMyc-Ub^K63^ and either dTAB2-V5 (wt) or dTAB2^ZnFDel^–V5 into *Drosophila* S2 cells. Cells were lysed 48 hrs post-transfection, immunoprecipitated with anti-HA antibody, resolved on 10% SDS PAGE and immunoblotted with anti-cMyc antibody. Observe that TAK1 is stabilised in the cell lysate in the presence of dTAB2^ZnFDel^ (dTAK1 panel left lane). Protein size markers (NEB) are depicted with values in kDa. (D) *Drosophila* Trabid binds strongly to dTAB2^ZnFDel^. S2 cells were transfected with dTrabid-HA alone or with either dTAB2^ZnFDel^ –V5 or dTAB2-V5. Cells were lysed 48 hrs post-transfection, immunoprecipitated with anti-V5 antibody, resolved on 10% SDS PAGE and immunoblotted with anti-HA antibody. Trabid bound very weakly to wild type TAB2 (middle lane). Observe the increase in binding of Trabid to dTAB2^ZnFDel^ (right lane) in comparison to wild type dTAB2.

We generated a dTAB2 mutant (dTAB2^ZnFDel^ see Figure 7A) where the first Cys residues in the C-terminal Zinc Finger (ZnF) motif were changed to Ala (C769A and C772A) as previously performed with human TAB2 [40]–[42]. Thereafter, the effect of this mutation on IMD signalling was assayed in cell culture using qPCR. *Drosophila* TAK1 was transfected either alone or together with dTAB2 or dTAB2^ZnFDel^ into S2 cells and *dipt* expression assayed 48 h post infection using qPCR. Interestingly, signalling intensity was doubled in the presence of dTAB2^ZnFDel^ when compared with wild type TAB2 (Figure 7B). Increased *dipt* induction may be the result of greater dTAK1 activation. Therefore the level of K63-linked ubiquitination of dTAK1 was examined in an S2 cell-based Ub assay in the presence of either TAB2 or dTAB2^ZnFDel^. Our results showed that, K63-linked ubiquitination of dTAK1 was increased in the presence of dTAB2^ZnFDel^ (Figure 7C, left lane). These results suggested therefore that the mutation of the dTAB2 ZnF domain might stabilise dTAK1 by enhancing its 63K-linked ubiquitination. This led to an increase in signalling capacity seen by increased *dipt* induction. It appeared therefore that the TAB2 C-terminal ZnF domain restricted dTAK1 activity and thereby the IMD signal. Interestingly, dTAB2^ZnFDel^ interacted more strongly (judging from the intensity of the signal) with dTrabid than wild type dTAB2 (Figure 7D). This might indicate that the tighter dTrbd bound to dTAB2 the higher the signalling capacity of dTAK1.

## Discussion

Our results point to a multi-tiered regulation of dTAK1 signalling in the IMD cascade. Ubiquitination played a key role in this regulation. It has been previously shown that in humans Lys158 was the Ub acceptor for K63 ubiquitination while Lys72 was the Ub acceptor for K48 ubiquitination [26]–[29]. In dTAK1 both Lys142 and Lys156 proved to be important for immune signalling as seen in qPCR assays in cell culture following transient transfection of these mutants. Our results indicate that in *Drosophila* TAK1, Lys142 functioned as the probable K63-acceptor site and Lys156 functioned as the probable K48-acceptor site. Our working hypothesis therefore, is that there is a main K63-Ub acceptor site in dTAK1 namely Lys142, which positively modulates the IMD immune signal. An interesting aspect of our results is that mutations in Lys142 and Lys156 do not affect the JNK pathway, in the transient activation of which TAK1 is involved. This suggests that AMP induction and JNK target activation can be independent in agreement with Silverman and co-workers [14]. In contrast to our results, Delaney *et al* [43] found that a TAK1 null mutation (*TAK1^179^*) in the kinase domain affected both pathways. Interestingly, the effect of *TAK1^179^* could be moderately rescued by overexpressing the JNK kinase Hemipterous [43]. Moreover, loss of function mitotic clones in the larval fat body of JNK pathway components downstream of TAK1 abolished expression of a *dipt-lacZ* marker [43]. One explanation for this difference could be that TAK1^K142R^ and TAK1^K156R^ may be important for the ubiquitination and activity status of TAK1 in regards to IMD pathway only-in contrast to *TAK1^179^*, which inactivates the kinase function presumably important for both IMD and JNK. However, ref 14 as well as this study conducted experiments in S2 cells, which limits analysis compared to the whole organism. An additional caveat of our experiments in cell culture was that they were conducted by transient transfection and overexpression of the relevant proteins in contrast to [14], which used stable cell lines. Transferring our TAK1 Lys142 and Lys156 mutants in flies and in a TAK1 mutant background will be able to solve this issue.

In our ubiquitination assays of dTAK1, a major band is seen at ∼84 kDa (coinciding with the molecular weight of monoubiquitinated dTAK1) and is visible when blotted with the antibody specific for Ub (Figure 2A; Figure 3A). Therefore, this band is likely to be the monoubiquitinated form of dTAK1. A similar band is observed in humans [26], [27]. Hence, it appears that a significant amount of total ubiquitinated dTAK1 is monoubiquitinated. Overexpression of dTAK1 alone is sufficient to activate the IMD pathway, as seen in previous studies [14] and in our qPCR assays. Even when expressed alone, *Drosophila* TAK1 immunoprecipitation shows ladder-like bands, indicative of mono and polyubiquitination (Figure 6C, see first lane). Therefore, we can assume that dTAK1 ubiquitin modification occurs even when overexpressed alone (with the aid of endogenous components).

Like K63 polyubiquitination, monoubiquitination is also implicated in degradation-independent functions including protein kinase activation, DNA repair, membrane trafficking and chromatin remodelling [reviewed in 44]. It was shown that chronic phosphorylation of IKKβ at Ser-177/Ser-181 leads to monoubiquitin attachment at nearby Lys-163, which in turn modulates the phosphorylation status of IKKβ during chronic inflammation [45]. Similarly, constitutive over-expression of dTAK1 may lead to its chronic activation and monoubiquitination. This may explain why singly overexpressed dTAK1 appears to have the same migration as a possibly monoubiquitinated form.

Trbd negatively regulated IMD signalling and reduced (the essential for activity) K63-linked ubiquitination of dTAK1, presumably through its DUB function. In the absence of infection, flies deficient in Trbd had a significant increase in steady-state expression of *dipt* (an IMD target) in their whole body. In addition, they exhibited induction over and above the wild type threshold following gut infection. This was not a consequence of delayed pathogen clearance as bacteria were cleared in *trbd* flies as fast as in wild type flies following both systemic and oral infection. Finally, the life span of *trbd* flies was dramatically reduced in the absence of immune challenge. This phenomenon became even more pronounced when another negative regulator of the IMD-mediated response (*pirk*) was also absent. This indicated the importance of negative regulators in shaping the immune reaction and highlighted the tight constraints, which IMD signalling is under inside the cell from the level of the receptor PGRP-LC through to Imd itself and the IIK complex. This reduction in life span was suppressed in a *dredd* mutant background indicating that it was the chronic over-activation of IMD signalling that was compromising long-term survival. Interestingly, the endogenous flora did not influence this phenomenon since germ-free *pirk; trbd* flies showed indistinguishable survival compared to normally reared *pirk; trbd* flies. This result showed that reduction in life span was flora-independent in contrast to mutations in PGRP-LB, affecting extracellular recognition [18] or in *big bang*, which disrupts septate junctions between gut cells [46]. Results from these *in vivo* experiments are consistent therefore, with the hypothesis that Trbd acts inside the cell to suppress Imd signalling.

Our work in cell culture suggests that this suppression happens at the level of dTAK1 attenuating the IMD signal and helping to maintain a transient and tightly regulated immune response. Both C518 (the putative catalytic cysteine) and the 3 tandem NZF domains of dTrbd were required for its function. However, both the qPCR data (Figure 7B) and ubiquitination assays (Figure 7C) indicated that while dTrbd significantly reduced dTAK1 K63-linked ubiquitination and subsequent signalling, it did not completely abrogate it, leaving the possibility that it functions either indirectly or in a partially redundant manner with another DUB. An alternative scenario however, may implicate the *in vivo* existence of unknown cofactor(s), which regulate Trbd and whose concentration was limiting in our cell culture over-expression experiments. Such co-factors have been described for A20 [reviewed in 47]. More work is needed to distinguish between these possibilities.

Contrary to the mammalian model where mutations in the ZnF domain of hTAB2 result in failure to activate TAK1, our results show that mutations in the C-terminal ZnF domain of dTAB2 provoke a significant enhancement of IMD signalling. Nevertheless, genetic evidence in *Drosophila* has shown unequivocally that loss of TAB2 leads to loss of TAK1 signalling capacity and therefore loss of the IMD signal [19]. However, our results point to a more refined relationship between the two proteins since the ZnF domain of TAB2 seemed to moderate TAK1 activity by restricting its signalling capacity. Hence, it is improbable that the ZnF domain of dTAB2 binds to and brings in the E3 ligase, which activates TAK1, as is the case in humans. One plausible scenario would be that dTAB2ZnFDel binds more strongly to Trbd than wild type dTAB2 (as suggested by Co-IP in Figure 7D) thus keeping Trbd away from its target (TAK1). An alternative explanation would be that dTAB2ZnFDel interacts more with TAK1 than wild type dTAB2 thus stabilising TAK1 and increasing its signalling capacity. However, we did not observe such an increase in our Co-IP experiments (Figure S9).

### Conclusion

Our working model is that of a tripartite relationship involving dTAK1, dTAB2 and Trabid. TAB2 is needed to activate TAK1 but through its ZnF domain it modulates TAK1 signalling and the TAB2-Trbd interaction. The latter is important for turning down the immune branch of TAK1 signalling and thereby the IMD pathway during gut epithelia responses and keeping IMD in check systemically in the absence of infection. Mutations in the dTAB2 ZnF domain enhance the TAB2-Trabid interaction and result in a more stable TAK1 presumably by keeping away Trbd from its target. An alternative scenario would be that through its ZnF domain TAB2 recruits an additional protein. This hypothesis would predict the presence of a protein that would act in concert with Trbd sharing some of its characteristics (e.g. DUB activity). More work is needed to distinguish between these two possibilities.

## Materials and Methods

### Cell culture and transfection

*Drosophila* S2 cells (Invitrogen) were maintained at 25°C in Schneider's *Drosophila* Medium (BioWhittaker/Lonza), supplemented with 10% heat-inactivated FBS and antibiotics 100 U/ml penicillin G and 100 µg/ml streptomycin sulfate – all Invitrogen). Cells were transfected with 2 ug plasmid DNA using Effectene Transfection Reagent (Qiagen) according to the manufacturer's protocol. Empty pAc5.1/HA-His vector was used to ensure equal amounts of DNA were delivered in each transfection.

#### Proteosomal inhibition and expression profile analysis of dTAK1, dTAK1^K142R^ & dTAK1^K156R^

Expression vectors encoding dTAK1-V5, dTAK1^K142R^-V5 & dTAK1^K142R^-V5 were transiently transfected into S2 cells. An aliquot of cells from these fractions were withdrawn 48 hrs post-transfection and frozen. This constituted the starting material (0 hrs). The remaining cells were incubated with 75 uM of MG132 (Enzo Lifesciences) for 8 hrs. All samples were lysed, resolved on 10% SDS PAGE and immunoblotted with anti V5 antibody (Invitrogen).

### Immunoprecipitation, immunoblotting and Ub assays

Cells were lysed 48 hrs post-transfection in RIPA buffer (Sigma-Aldrich) supplemented with Complete Mini Protease Inhibitor Cocktail tablets (Roche Applied Science) and Benzonase Nuclease (Sigma-Aldrich). Cell lysates were incubated rocking with 50 µl of Anti-V5 or Anti-HA Agarose Affinity Gel (Sigma-Aldrich) for 2 hours at 4°C. Antibody beads were pre-blocked in RIPA buffer supplemented with 0.2% BSA (NEB) at 4°C for 2 hrs. Immunoprecipitates were washed with 600 µl CoIP wash buffer 900 (50 mM Tris-HCl [pH 8.0], 900 mM NaCl, 5 mM EDTA [pH 8.0], 0.5% Igepal CA-6030) four times for 10 minutes each at room temperature, followed by a final wash with 600 µl CoIP wash buffer 150 (50 mM Tris-HCl [pH 8.0], 150 mM NaCl, 5 mM EDTA [pH 8.0], 0.5% Igepal CA-6030). Immunoprecipitates were eluted in 1X SDS sample buffer, resolved on 10% SDS-PAGE and transferred to PVDF membranes. Blots were probed with mouse anti-V5 (1∶5000, Invitrogen), mouse anti-c-Myc peroxidise (1 µg.ml^−1^.Roche Applied Science; Clone 9E10) or rat anti-HA antibodies (200 ng.ml^−1^, Roche Applied Science; Clone 3F10). Between probing with a different antibody, blots were stripped with Restore PLUS Western Blot Stripping Buffer (Pierce).

#### Quantitative PCR analysis in S2 cells

Total RNAs were extracted from 10^6^ S2 cells/ml using TRIzol (Invitrogen). Total RNA was treated with DNase 1 (Roche) and cDNA was prepared from 1 µg total-RNA using AffinityScript Multiple Temperature cDNA Synthesis Kit (Agilent). Triplicate cDNA samples were amplified with the Brilliant 11 SYBR Green QPCR Master Mix Kit (Agilent) in a Corbet Rotor-Gene 6000 QPCR machine (Qiagen) according to the manufacturer's protocols.

#### Quantitative PCR analysis in adult flies

Total RNAs were extracted from whole flies (10♀) using Total RNA Purification Plus Kit (Norgen – Biotek) and cDNA was prepared from 0.5 µg total RNA using Maxima First Strand cDNA Syntesis Kit (ThermoScientific). Triplicate cDNA samples were amplified with the SensiFASAT SYBR No-ROX Kit (Bioline) in a Corbet Rotor-Gene 6000 QPCR machine (Qiagen) according to the manufacturer's protocols.

For both cells and adults experiments were performed at least three times independently, giving similar results. All transcript expression values were normalized to actin and were quantified relative to a control using the ΔΔCt method. Following primers were used for QPCR amplification, Upd3 and Soc36E primers as in [18], [33]:

*attacinA*; F 5′-CTCCTGCTGGAAAACATC-3′;

R: 5′-GCTCGTTTGGATCTGACC-3′;

*cecropinA1*; F: 5′- CATTGGACAATCGGAAGCTGGGTG-3′;

R: 5′- TAATCATCGTGGTCAACCTCGGGC-3′;

*diptericin*; F: 5′- CCGCAGTACCCACTCAATCA-3′;

R: 5′- TGTGATCTGCAGGATGGTGT-3′;

*drosocin*; F: 5′- GTTCACCATCGTTTTCC -3′;

R: 5′- CCACACCCATGGCAAAAAC -3′;

*puckered*;- F: 5′- CATCATCAACGGCAATGAAC -3′;

R: 5′- TTGGGACTTTGGCAGGTAAC -3′;

*Rp49*; F 5′- CCAGTCGGATCGATATGCTAA-3′;

R: 5′- GTTCGATCCGTAACCGATGT-3′;

#### *Drosophila* stocks and infection experiments

The following stocks were used: the *trbd* deletion mutant strain generated by homologous recombination [30], the *yw* strain (used as the wild type and for being the genetic background the *trbd* deletion was generated), *pirk^EY00723^* [21], *Dredd^B118^* [9] as the negative control and to disable the IMD pathway in a *trbd* or *pirk; trbd* background. Bacterial strains used were the Gram-negative bacteria *Erwinia carotovora carotovora 15 (Ecc15)* and *Escherichia coli 1106 (Ec1106)*.

Systemic bacterial infections were performed using a micro-injector (Drummond Scientific Nanoject II) coupled to a fine glass needle. Bacteria were harvested by centrifugation (4000 rpm for 4 minutes) and washed in phosphate saline buffer (1× PBS). Washed cells were again centrifuged and resuspended in PBS to an OD600 of 0.25 (measured with a Thermo Scientific Nanodrop 1000 spectrophotometer). To infect flies, 13.8 nl from the above inoculant (≈4000 cells),or from a 10 fold dilution with PBS (≈400 cells) was injected.

Oral bacterial infections were performed on female flies (2–4 days old) following a 2 h starvation at 29°C, by applying a concentrated bacterial solution *Ecc15* (OD = 200 at 600 nm) supplemented with sucrose (final concentration 5%) to a filter disk placed on the surface of the medium of the vial. Flies were infected at 29°C for 24 h, then flipped to a fresh fly food vial and maintained at 29°C [18].

Bacterial persistence for oral infections were evaluated at 24 hrs post-infection in triplicates by crushing 3 cohorts of 10 flies for each repeat in Luria Broth (LB) culture medium. Bacterial counts for systemic infections were evaluated at 6 and 24 hrs post-infection in triplicates by crushing groups of 6 flies for each repeat in LB. Serial dilutions of these extracts were platted in triplicate in LB-agar and incubated overnight at 29°C.

#### Life-span studies, production and maintenance of germ-free flies

For life-span studies, cohorts of 30 flies (15♂+15♀) were put in vials and were monitored for their survival in 12 biological repeats (n = 360 per strain). The flies were tipped to fresh food every two days. Germ free flies were produced as previously described [48] and checked every five days for bacterial contamination by performing PCR analysis on fly homogenates using 16S eubacterial primers (63F//1387R) as well as by culturing the homogenates on LB plates.

#### DNA constructs and mutagenesis

*Drosophila tak1, trabid and tab2* were PCR amplified from the *D. melanogaster* complementary cDNA (DGRC, Indiana, USA; GenBank numbers AAF50895, AAF54429 and AAF57580) and cloned into pAc5.1/V5-His (Invitrogen) or pAc5.1/HA-His. Plasmid pAc5.1/HA was generated by replacing the V5 epitope of plasmid pAc5.1/V5-His (Invitrogen) with an HA epitope by ligating the following annealed phosphorylated oligomers into *Bst*BI-*Age*I-digested pAc5.1/V5-His:- HA-S: 5′-CGAAAGATCTGCATACCCATACGACGTCCCAGACTACGCTCGTA-3′; HA-AS: 5′-CCGGTACGAGCGTAGTCTGGGACGTCGTATGGGTATGCAGATCTTT-3′.

Ubiquitin was PCR amplified from pUAS–ubiquitin (kind gift from Prof Toshiro Aigaki) and N-terminally tagged with primers containing cMyc sequence and cloned into pAc5.1/HA-His. Point mutations were carried out using QuikChange Lightning Site-Directed Mutagenesis Kit and QuikChange Lightning Multi Site-Directed Mutagenesis Kit (Agilent) according to manufacturer's protocols. Mutagenesis primers were designed using the QuikChange Primer Design Application found in the Agilent website (www.genomics.agilent.com). A Mastercycler pro thermal cycler (Eppendorf) was used for PCR amplification. After amplification the parental (non-mutated) dsDNA strands were digested by addition of 2 µl Dpn 1 enzyme (for single site mutagenesis or 1 µl for multi-site mutagenesis) and incubating at 37°C for 5 mins. Thereafter, the mutated plasmids were transformed into XL10-Gold Ultra competent Cells.

The following point mutant constructs were made:

- ***Drosophila*** **Trabid**: dTrbd^C518S^ & dTrbd^C518S+3xNZFDel^ (first Cys residue of the 3 NZF domains, C13, C94 and C238, were mutated to Ala in addition to C518S).
- ***Drosophila*** **TAK1**: dTAK1^K134R^, dTAK1^K142R^, dTAK1^K156R^, dTAK1^K189R^ & dTAK1^K194R^
- ***Drosophila*** **TAB2**: dTAB2^ΔZnF^ (mutating the first 2 Cys residues in the C-terminal ZnF motif - C769A and C772A)
- **Ubiquitin**: cMyc-Ub^K48^ (mutation of all Lys sites to Arg except K48) & cMyc-Ub^K63^ (mutation of all Lys sites to Arg except K63).

#### Statistical analysis

All statistical analyses were performed using Prism 6 (GraphPad software). Lifetime data were analysed as in [49]. Briefly, we pooled the lifespan data (n = 360) from our six independent biological repeat sets of life span assays for each genotype (i.e. there were no significant departures between repeats; P>0.1, log-rank test) and plotted survival curves for each *Drosophila* strain using Kaplan Meier estimates. Significant differences between survival data of different genotypes (or different treatment of the same genotype) were identified using Log Rank and Wilcoxon tests (ChiSquare and p-values).

For qPCR analysis, we performed three biologically independent measurements per combination of constructs (Figure 1B; Figure 6B; Figure 7B; Figure S1; Figure S3B; Figure S7) or genotypes (Figures 4B–E; Figure S6). All AMPs mRNA values were normalised to RP49 and expressed relative to the empty vector or the *yw* strain. All values were expressed as mean values and were plotted with standard error. Student's t-test (Figure 1B; Figure 4B; Figure 4E; Figure 6B; Figure 7B, Figure S1; Figure S3B; Figure S6; Figure S7) or paired t-test (Figure 4C; Figure 4D) were employed to ascertain statistical significance.

## Supporting Information

Figure S1 Influence of Lys 142, 134, 156, 189, 194 on TAK1 activity. (A) Only mutating TAK1 Lys 142 or 156 influenced *dipt* expression (read-out for IMD) following transient transfection. (B) However, Lys 142 and 156 had no effect on *puc* (used as a read-out for JNK).
(TIF)

Figure S2 Expression profiles of Lys 142 and 156 mutants of *Drosophila* TAK1. Both dTAK1^K142R^ & dTAK1^K156R^ show similar expression profiles to dTAK1. A time-course expression analysis after treatment with proteasomal inhibitor MG132 (75 µM for 8 hrs) show the profiles of both mutants were similar to full-length dTAK1.
(TIF)

Figure S3 A time course profile of endogenous TAK1 ubiquitination in S2 cells. (A) We co-transfected cMyc-Ub^K63^ or cMyc-Ub^K48^ with dTAB2-HA followed by challenge with *E. coli* peptidoglycan (PG). Immunoprecipitation with an antibody against endogenous TAK1 and blotting with cMyc revealed a bias towards 63K-linked ubiquitination 2 h post challenge that gradually shifted towards 48K-linked ubiquitination 6 h post challenge. (B) Triggering of IMD was statistically indistinguishable when dTAK1 was transiently transfected and when PG was added to cells.
(TIF)

Figure S4 Bacterial persistence in Trabid mutants after Ecc15 oral infection. Bacterial persistence in *yw, pirk, pirk;trbd, trabd/+* and *trabd* after oral infection with *Ecc15*. *Ecc15* colony-forming units (CFUS) from homogenates of 10 flies were counted. Data correspond to six independent experiments. No statistically significant values were found between any of the strains using a Student's t test.
(TIF)

Figure S5 Bacterial persistence in Trabid mutants after Ec1106 and Ecc15 systemic infection. Bacterial persistence in *yw, pirk, pirk;trbd, trabd/+ trabd* and *dredd* after systemic infection (injection) with *Ec1106* or *Ecc15*. Colony-forming units (CFUs) from homogenates of six flies were counted for two different time points 6 and 24 hrs following infection using two initial bacterial loads (low dose≈400 cells, high dose≈4000 cells). Each data set corresponds to three independent experiments. Bacterial clearance in *trabd* and *pirk; trbd* was statistically faster than *yw* control and *pirk* flies using either low or high dose of *Ec1106* (Fig. S5A and S5B respectively) using a Student's t-test. Following *Ecc15* systemic infection with the same doses, the differences between *trbd* mutants and *yw* were just below the limit of statistical significance using a Student's t-test (Fig. S5C and S5D). *p<0.05, **p<0.01, ***p<0.001, ****p<0.0001; x = no flies alive.
(TIF)

Figure S6 Chronic disruption of gut homeostasis in *trbd* mutants. (A) *Upd3* and (B) *Socs36E* was significantly elevated in guts from *trbd* (*) and *pirk;trbd* (**) flies in comparison to wild type (*yw*) and *dredd; pirk;trbd* flies as determined by Student's t-test (p<0.005 in both cases). Values are mean values from three independent experiments with standard error. Measurements were taken 7 days post eclosion.
(TIF)

Figure S7 Trbd does not influence JNK-related activity of TAK1. Co-transfection of Trbd with TAK1 or TAK1+TAB2 showed that expression of *puc* was statistically indistinguishable between TAK1 and TAK1+dTrbd or TAK1+TAB2 and TAK1+TAB2+dTrbd.
(TIF)

Figure S8 *Drosophila* Trabid does not affect K48-linked polyubiquitination in dTAK1. Expression vectors containing C-terminally HA-tagged dTrabid, dTrabidC518S or dTrabidC518S+3xNZFDel, along with dTAK1-V5, dTAB2-HA and cMyc-UbK48 were co-transfected in S2 cells in the combinations shown. Cells were lysed 48 hrs post-transfection, immunoprecipitated with anti-V5 antibody, resolved on 10% SDS PAGE and immunoblotted with anti-cMyc peroxidise. Protein size markers (NEB) are depicted adjacent to top panels with values given in kDa.
(TIF)

Figure S9 dTAB2^ZnFDel^ does not bind more to dTAK1 than wild type dTAB2. S2 cells were transfected with dTAK1-HA with either dTAB2^ZnFDel^ –V5 or dTAB2-V5. Cells were lysed 48 hrs post-transfection, immunoprecipitated with anti-V5 antibody, resolved on 10% SDS PAGE and immunoblotted with anti-HA antibody. TAK1 bound to wild type TAB2 (left lane) as well to dTAB2^ZnFDel^ –V5.
(TIF)
