# Supplementary figures and images for: Loss of Trabid, a New Negative Regulator of the Drosophila Immune-Deficiency Pathway at the Level of TAK1, Reduces Life Span
Source: PLoS Genet. 2014 Feb 20;10(2):e1004117. doi: 10.1371/journal.pgen.1004117 (PMC3930493; doi:10.1371/journal.pgen.1004117)

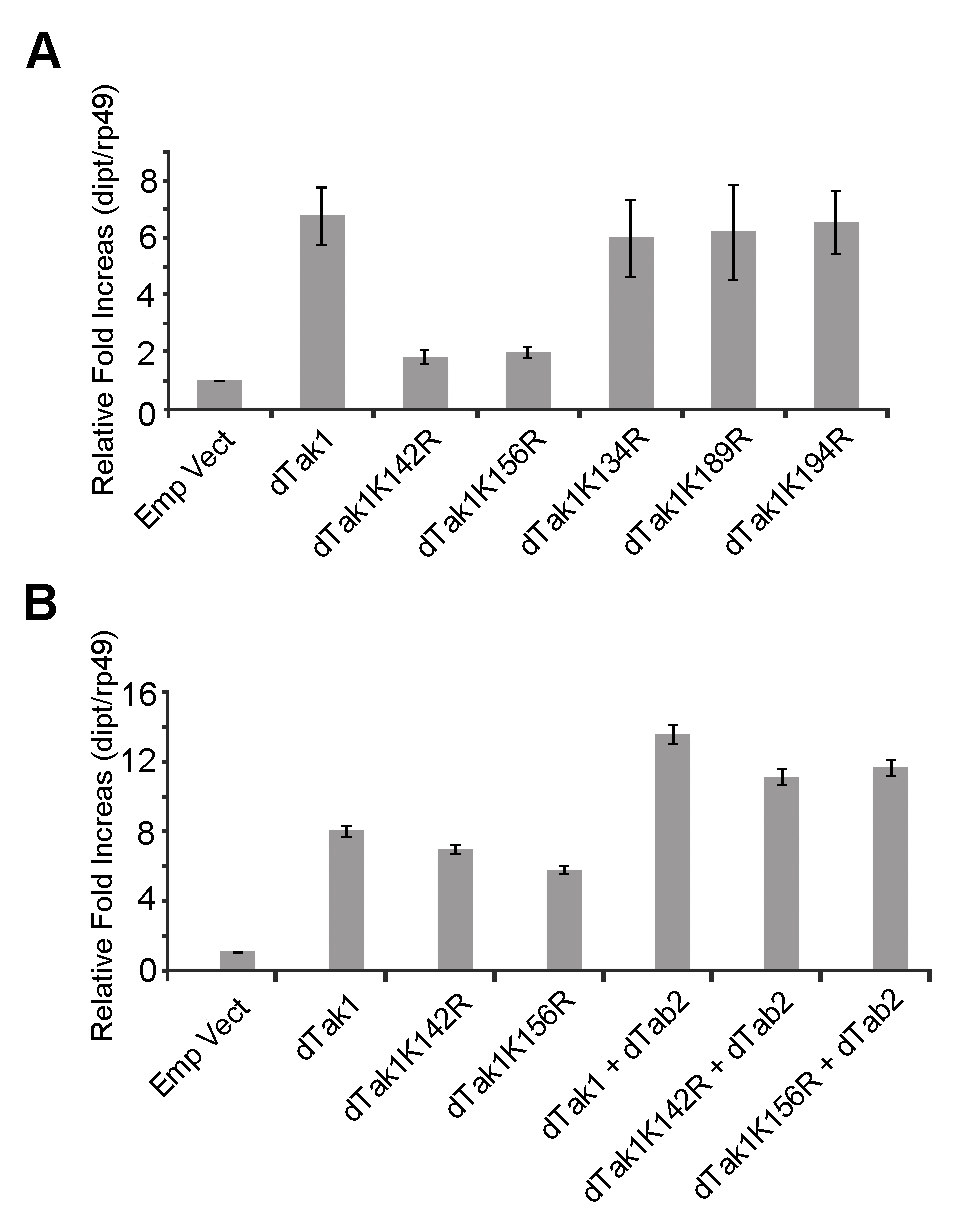

Supplement: Figure S1 — Influence of Lys 142, 134, 156, 189, 194 on TAK1 activity. (A) Only mutating TAK1 Lys 142 or 156 influenced dipt expression (read-out for IMD) following transient transfection. (B) However, Lys 142 and 156 had no effect on puc (used as a read-out for JNK). (TIF) [file pgen.1004117.s001.tif]

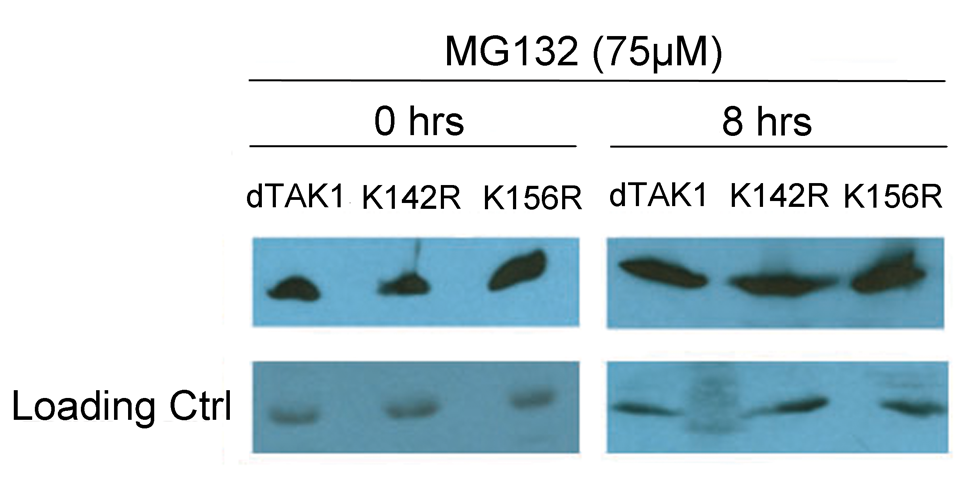

Supplement: Figure S2 — Expression profiles of Lys 142 and 156 mutants of Drosophila TAK1. Both dTAK1K142R & dTAK1K156R show similar expression profiles to dTAK1. A time-course expression analysis after treatment with proteasomal inhibitor MG132 (75 µM for 8 hrs) show the profiles of both mutants were similar to full-length dTAK1. (TIF) [file pgen.1004117.s002.tif]

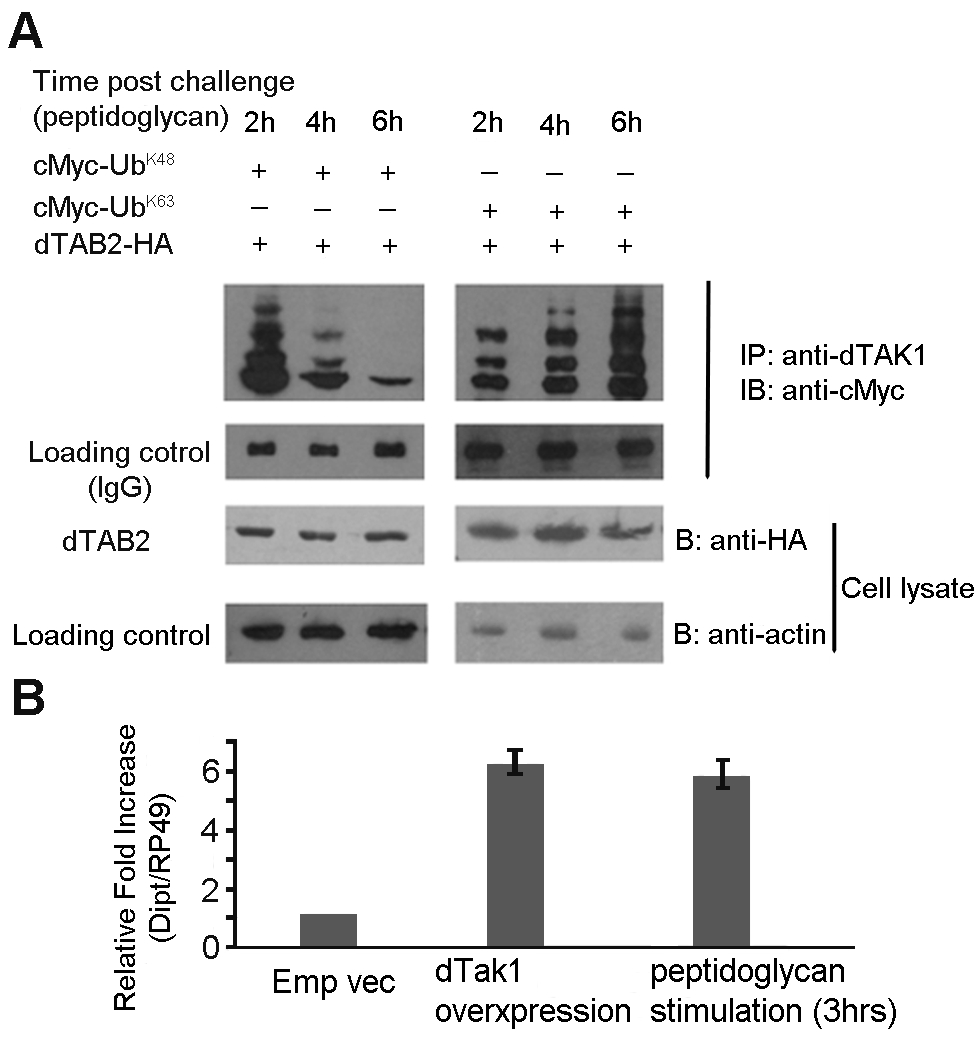

Supplement: Figure S3 — A time course profile of endogenous TAK1 ubiquitination in S2 cells. (A) We co-transfected cMyc-UbK63 or cMyc-UbK48 with dTAB2-HA followed by challenge with E. coli peptidoglycan (PG). Immunoprecipitation with an antibody against endogenous TAK1 and blotting with cMyc revealed a bias towards 63K-linked ubiquitination 2 h post challenge that gradually shifted towards 48K-linked ubiquitination 6 h post challenge. (B) Triggering of IMD was statistically indistinguishable when dTAK1 was transiently transfected and when PG was added to cells. (TIF) [file pgen.1004117.s003.tif]

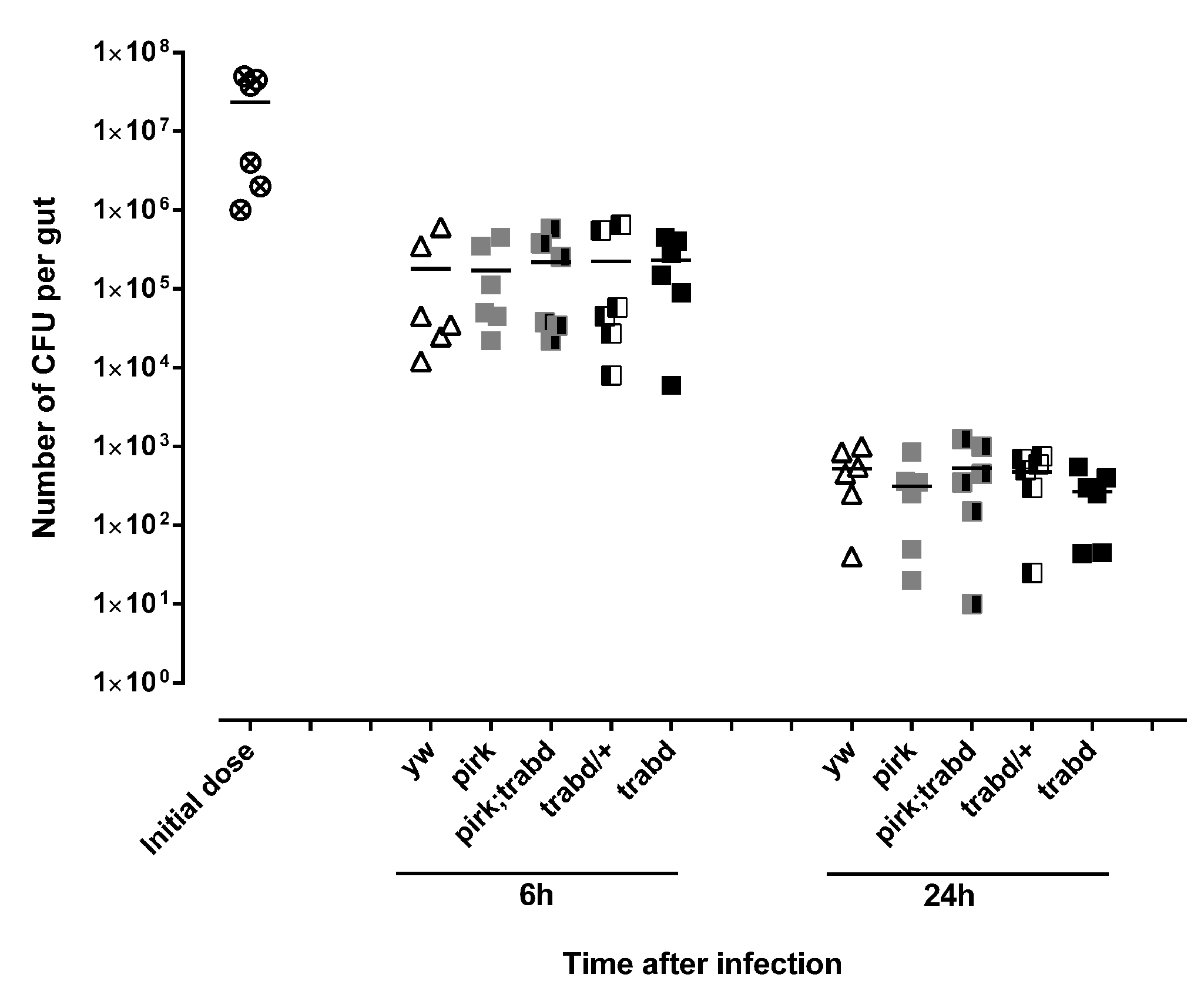

Supplement: Figure S4 — Bacterial persistence in Trabid mutants after Ecc15 oral infection. Bacterial persistence in yw, pirk, pirk;trbd, trabd/+ and trabd after oral infection with Ecc15. Ecc15 colony-forming units (CFUS) from homogenates of 10 flies were counted. Data correspond to six independent experiments. No statistically significant values were found between any of the strains using a Student's t test. (TIF) [file pgen.1004117.s004.tif]

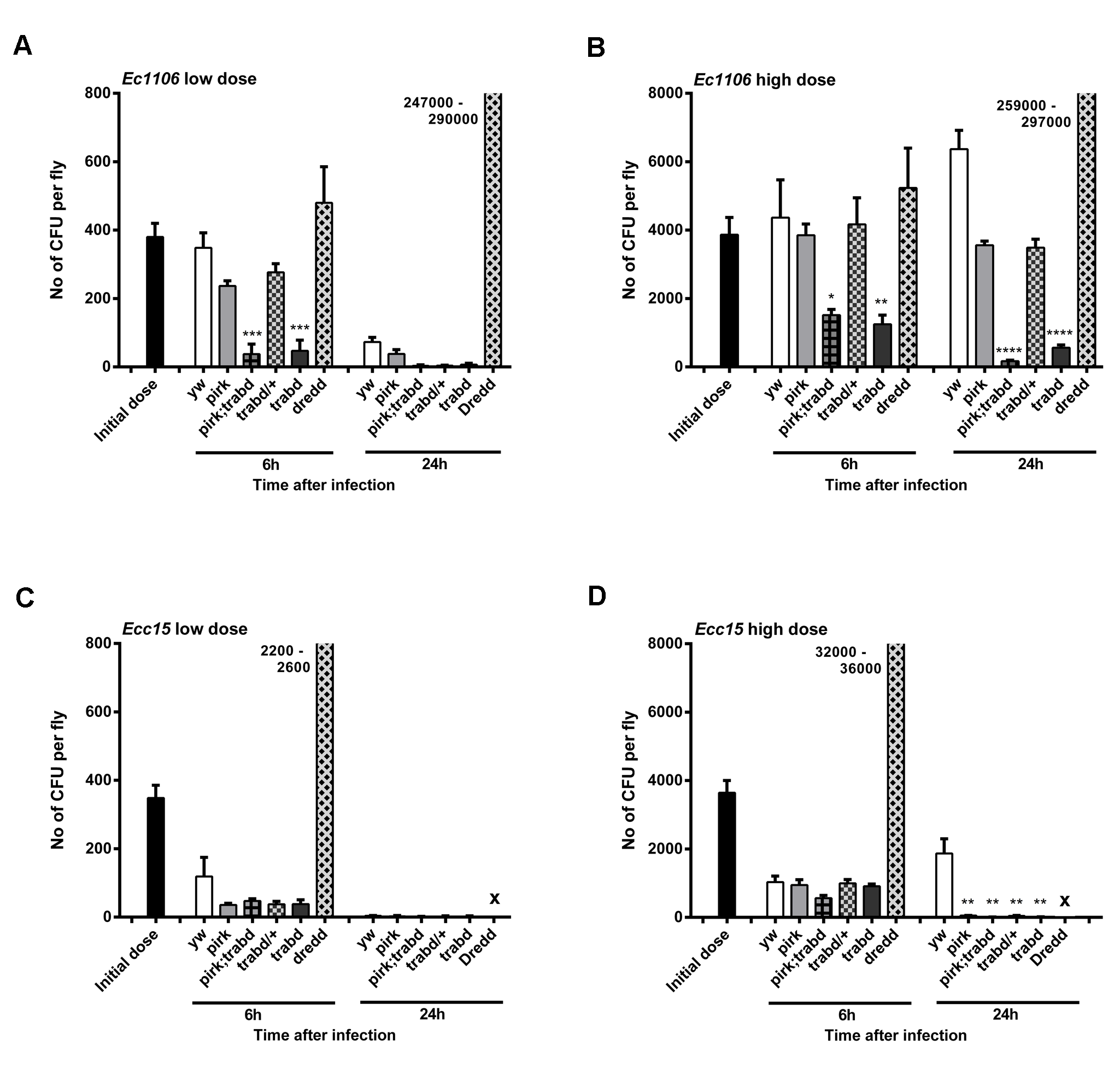

Supplement: Figure S5 — Bacterial persistence in Trabid mutants after Ec1106 and Ecc15 systemic infection. Bacterial persistence in yw, pirk, pirk;trbd, trabd/+ trabd and dredd after systemic infection (injection) with Ec1106 or Ecc15. Colony-forming units (CFUs) from homogenates of six flies were counted for two different time points 6 and 24 hrs following infection using two initial bacterial loads (low dose≈400 cells, high dose≈4000 cells). Each data set corresponds to three independent experiments. Bacterial clearance in trabd and pirk; trbd was statistically faster than yw control and pirk flies using either low or high dose of Ec1106 (Fig. S5A and S5B respectively) using a Student's t-test. Following Ecc15 systemic infection with the same doses, the differences between trbd mutants and yw were just below the limit of statistical significance using a Student's t-test (Fig. S5C and S5D). *p<0.05, **p<0.01, ***p<0.001, ****p<0.0001; x = no flies alive. (TIF) [file pgen.1004117.s005.tif]

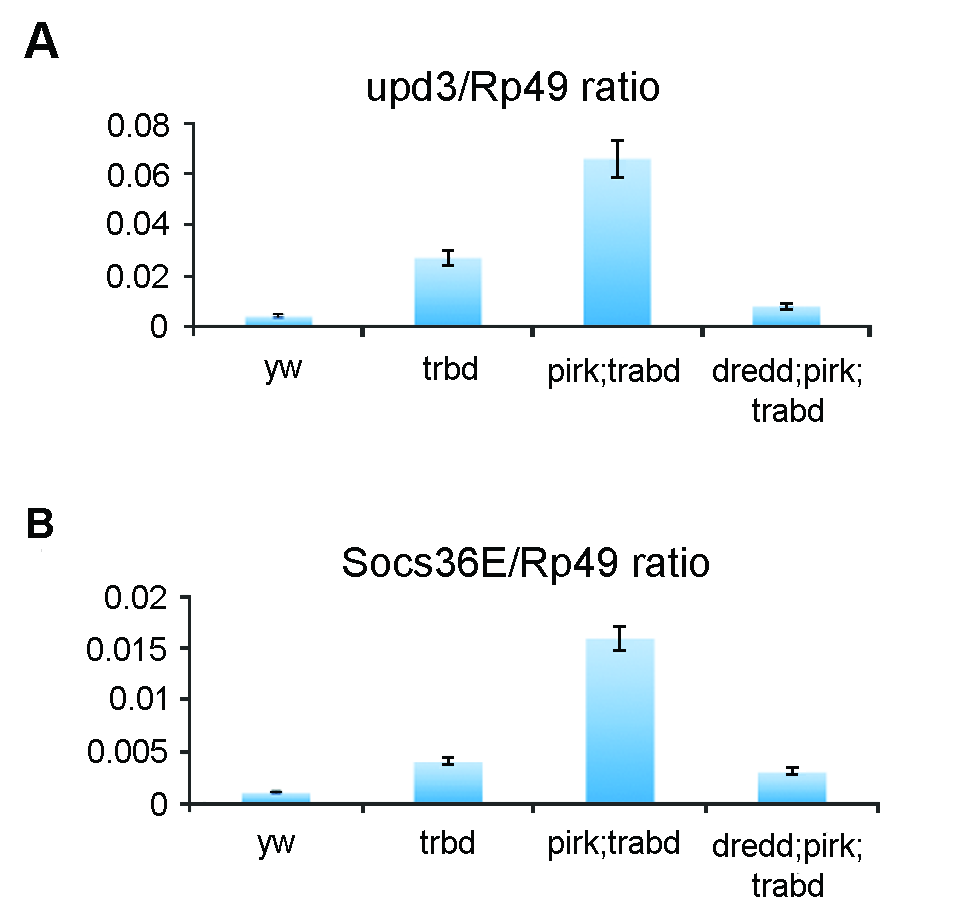

Supplement: Figure S6 — Chronic disruption of gut homeostasis in trbd mutants. (A) Upd3 and (B) Socs36E was significantly elevated in guts from trbd (*) and pirk;trbd (**) flies in comparison to wild type (yw) and dredd; pirk;trbd flies as determined by Student's t-test (p<0.005 in both cases). Values are mean values from three independent experiments with standard error. Measurements were taken 7 days post eclosion. (TIF) [file pgen.1004117.s006.tif]

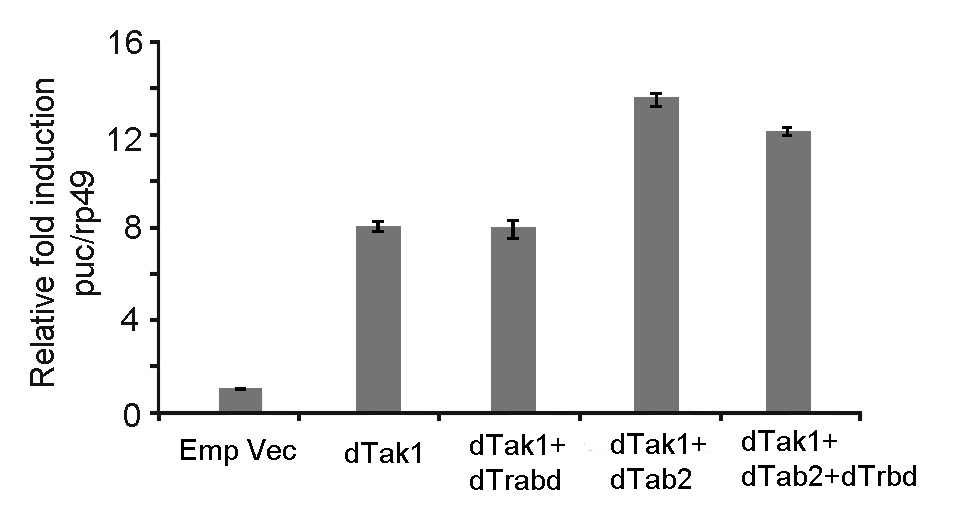

Supplement: Figure S7 — Trbd does not influence JNK-related activity of TAK1. Co-transfection of Trbd with TAK1 or TAK1+TAB2 showed that expression of puc was statistically indistinguishable between TAK1 and TAK1+dTrbd or TAK1+TAB2 and TAK1+TAB2+dTrbd. (TIF) [file pgen.1004117.s007.tif]

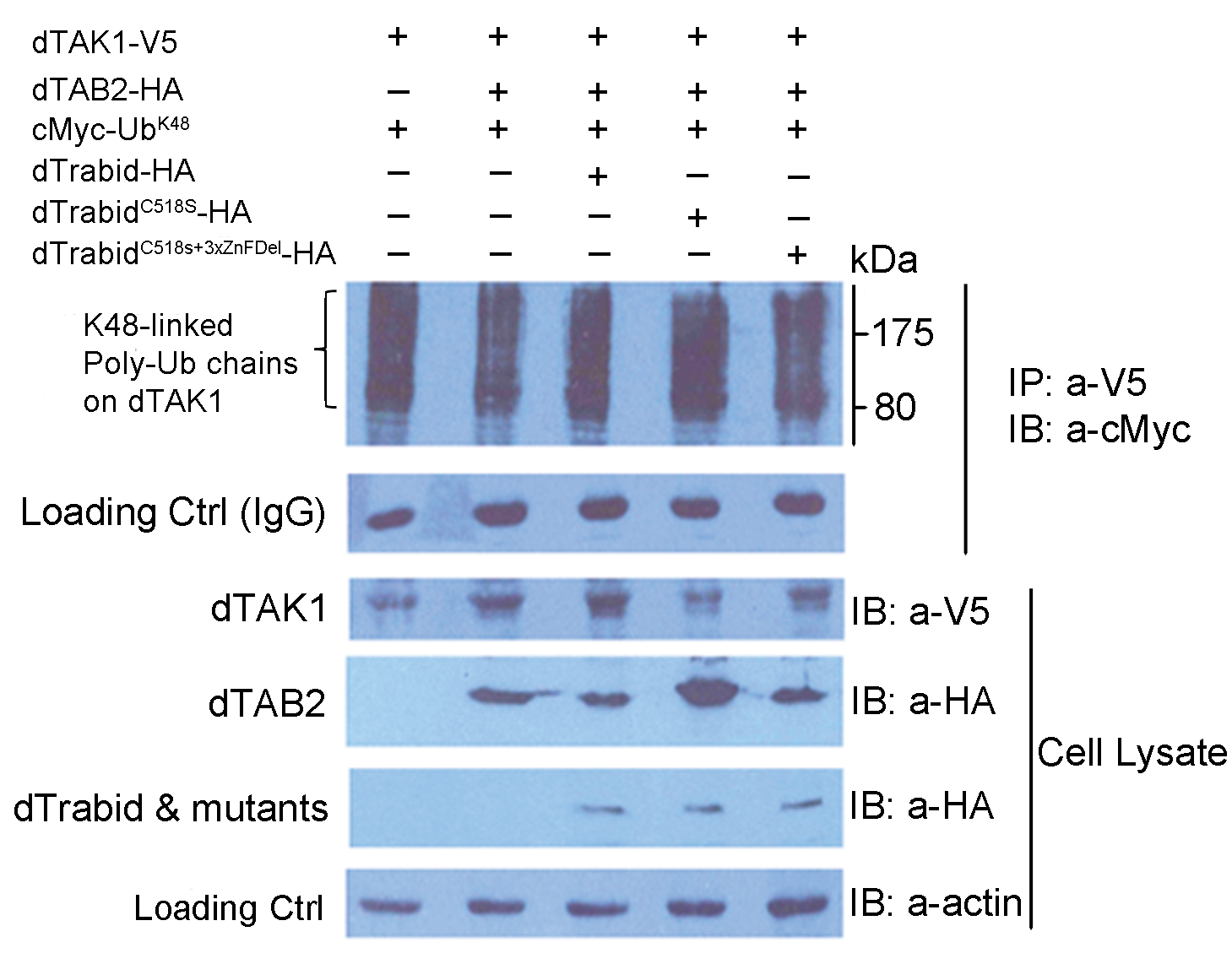

Supplement: Figure S8 — Drosophila Trabid does not affect K48-linked polyubiquitination in dTAK1. Expression vectors containing C-terminally HA-tagged dTrabid, dTrabidC518S or dTrabidC518S+3xNZFDel, along with dTAK1-V5, dTAB2-HA and cMyc-UbK48 were co-transfected in S2 cells in the combinations shown. Cells were lysed 48 hrs post-transfection, immunoprecipitated with anti-V5 antibody, resolved on 10% SDS PAGE and immunoblotted with anti-cMyc peroxidise. Protein size markers (NEB) are depicted adjacent to top panels with values given in kDa. (TIF) [file pgen.1004117.s008.tif]

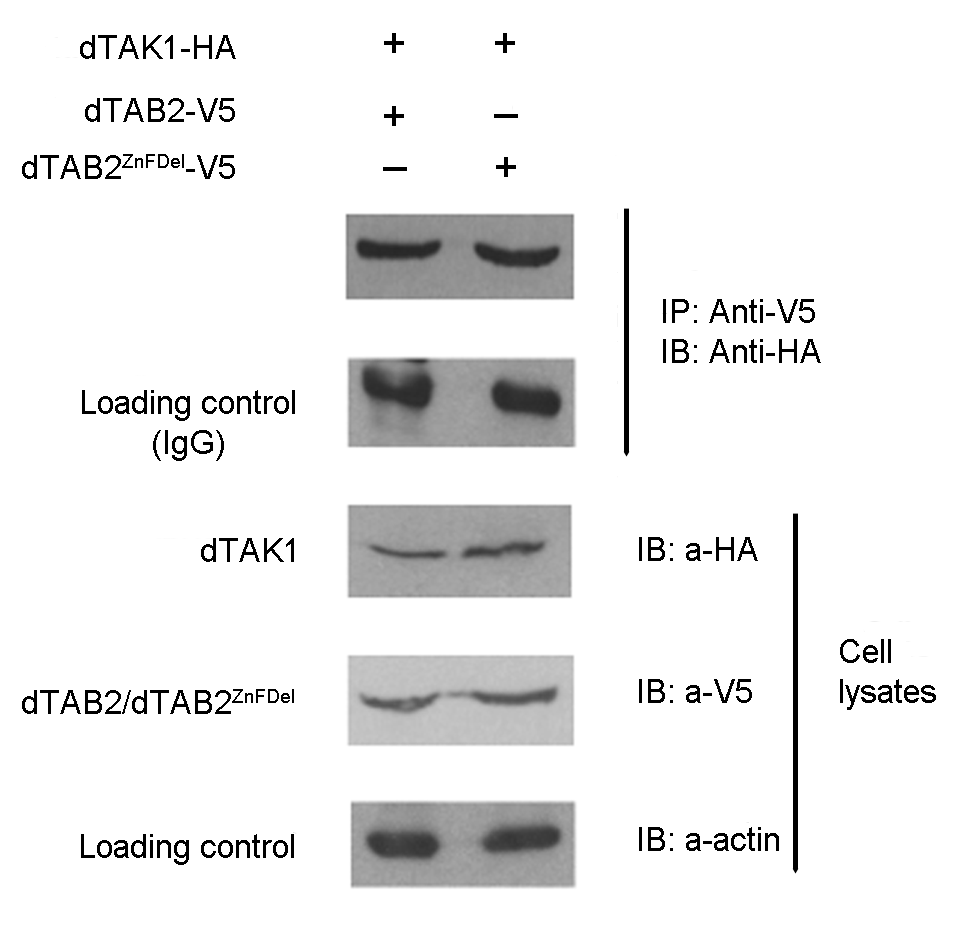

Supplement: Figure S9 — dTAB2ZnFDel does not bind more to dTAK1 than wild type dTAB2. S2 cells were transfected with dTAK1-HA with either dTAB2ZnFDel –V5 or dTAB2-V5. Cells were lysed 48 hrs post-transfection, immunoprecipitated with anti-V5 antibody, resolved on 10% SDS PAGE and immunoblotted with anti-HA antibody. TAK1 bound to wild type TAB2 (left lane) as well to dTAB2ZnFDel –V5. (TIF) [file pgen.1004117.s009.tif]
